# Bacterial synergies amplify nitrogenase activity in diverse systems

**DOI:** 10.1093/ismeco/ycae158

**Published:** 2024-12-12

**Authors:** Andrew W Sher, Robert J Tournay, Emma Gomez-Rivas, Sharon L Doty

**Affiliations:** School of Environmental and Forest Sciences, College of the Environment, University of Washington, Seattle, WA 98195-2100, United States; School of Environmental and Forest Sciences, College of the Environment, University of Washington, Seattle, WA 98195-2100, United States; Department of Microbiology, University of Washington, Seattle, WA 98195, United States; School of Environmental and Forest Sciences, College of the Environment, University of Washington, Seattle, WA 98195-2100, United States

**Keywords:** endophytes, nitrogen fixation, microbial interactions, populus, symbiosis

## Abstract

Endophytes are microbes living within plant tissue, with some having the capacity to fix atmospheric nitrogen in both a free-living state and within their plant host. They are part of a diverse microbial community whose interactions sometimes result in a more productive symbiosis with the host plant. Here, we report the co-isolation of diazotrophic endophytes with synergistic partners sourced from two separate nutrient-limited sites. In the presence of these synergistic strains, the nitrogen-fixing activity of the diazotroph is amplified. One such partnership was co-isolated from extracts of plants from a nutrient-limited Hawaiian lava field and another from the roots of *Populus* trees on a nutrient-limited gravel bar in the Pacific Northwest. The synergistic strains were capable of increasing the nitrogenase activity of different diazotrophic species from other environments, perhaps indicating that these endophytic microbial interactions are common to environments where nutrients are particularly limited. Multiple overlapping mechanisms seem to be involved in this interaction. Though synergistic strains are likely capable of protecting nitrogenase from oxygen, another mechanism seems evident in both environments. The synergies do not depend exclusively on physical contact, indicating a secreted compound may be involved. This work offers insights into beneficial microbial interactions, providing potential avenues for optimizing inocula for use in agriculture.

## Introduction

As conceptual understanding has moved from isolated individual species to metaorganisms of diverse and interacting species, the importance of both microbe–host and microbe–microbe interactions in ecology has been recognized. Genomic analyses have revealed that microbial metabolic exchange is hardwired, with amino acid biosynthesis optimized to support synergistic growth, reducing individual metabolic burden [1]. In natural systems, microorganisms commonly form multispecies communities, communicating and cooperating with the exchange of signals and resources [2]. Detailed studies of these interactions can reveal new insights even for long-known symbiosis such as lichens, thought to consist only of a mycobiont and a photobiont but recently shown to consist of other key players, Basidiomycete yeasts and bacteria [3]. Root nodules of legumes long thought to house only one specific nitrogen (N)-fixing rhizobial strain is now known to also contain non-N-fixers that can promote plant growth in other ways [4]. Despite the powerful impact of the microorganisms within the plant microbiome on the host plant, such as providing tolerances against abiotic and biotic stresses as well as promoting plant growth and health, little is known about how microbe–microbe interactions affect or direct these impacts (reviewed in [5]).

Through a reductionist approach, synthetic communities can be used to begin to understand microbial interactions and the potential metabolic cross-feeding that can enable novel or improved activities to develop. In an artificial system with a cyanobacterial strain and a yeast strain, for example, the growth rate of the yeast doubled compared to its growth in isolation [6]. It was shown that the alga provided 10 metabolites and oxygen to the system while the fungus provided carbon dioxide and leucine. In a study of a synthetic community consisting of 16 strains from the rhizosphere of switchgrass, a few strains quickly dominated, requiring adjustment of strain ratios to maximize the alpha diversity [7]. Similarly, in a study on phyllosphere microbiota interactions in *Arabidopsis*, 90% of the interactions were negative, suggesting that the interactions are mostly competitive [8]. A synthetic community of 10 taxonomically diverse strains from the rhizosphere of *Populus deltoides* grown in different media resulted in a few dominant strains, depending on the media used [9]. The *Pseudomonas* strain dominated the communities in both media, having negative interactions with the majority of the other strains. Few studies have investigated at a mechanistic level the interactions between endophytes, the microorganisms within plants. Co-occurrence relationships in these microbial networks indicate that positive correlations may dominate [10]. Cooperative interactions among plant microbiota can promote biofilm formation, molecular communication, dispersal, and nutritional interdependencies for access to nutrient-poor environments [10].

Biological nitrogen fixation (BNF) is a particularly energy-intensive process, yet it is an essential activity for life on Earth. For the reduction of each molecule of dinitrogen gas to ammonia, 8 electrons and at least 16 molecules of ATP are required as well as sufficient reducing power. The nitrogenase enzyme is acutely sensitive to oxygen [11], and managing oxygen may be a more energetically costly activity than nitrogen fixation itself [12]. The well-studied aerobic N-fixer (diazotroph), *Azotobacter vinelandii*, uses multiple mechanisms. These include respiratory protection where it rapidly oxidizes sugars and depletes intracellular oxygen [13], production of extracellular polymeric substances, increased cell size, and formation of a capsule around the cells [14]. The demanding nature of BNF seems to limit sufficient activity to a subset of capable strains. In microbial mats, for example, only a small percentage of the diazotrophic cells were actively fixing N_2_ [15]. In a study of an aerobic diazotroph of poplar (*Po. trichocarpa*), only 10% of the cells of a single strain were expressing nitrogenase and actively fixing nitrogen [16]. The metabolic signatures of the nitrogenase active and inactive sectors differed, with the N-fixing cells producing amino acids and polyamines, while the non-fixing cells had more trehalose and citric acid, potentially suggesting metabolic cross-feeding.

However, diazotrophs are rarely in isolation in nature. Other species in a microbial community may support BNF of the diazotrophic species. In a study of endophytic strains from maize xylem sap, two strains were identified as strong N-fixers [17]. Non-fixing strains from the xylem sap community were mixed with the diazotrophs, leading to the discovery that two of the strains, an *Acinetobacter* and a *Rosenbergiella epipactidis,* increased the nitrogenase activity of the diazotrophs. It was suggested that these helper strains created a micro-oxygen environment since incubation in 1% oxygen eliminated the need for the helper strains.

In this study, we isolated N-fixing endophytic strains from plants growing in N-limited environments, one from a Hawaiian lava field and one from a cobble-dominated riparian zone. In both environments, diazotrophic strains co-isolated with nondiazotrophs that amplified nitrogenase activity. We tested the specificity of the synergy effect on other diazotrophic strains and found that while all the synergistic strains increased the activity of most diazotrophic strains, some partnerships performed better than others. Moving beyond proof of principal, we then explored potential mechanisms of the synergy effect. Incubating the diazotrophs with reduced levels of oxygen did not substitute for the presence of the helper strain for two of three diazotrophs in our study, indicating that a mechanism other than oxygen reduction is at play. Physically separating these two diazotrophic strains from the partners did not prevent the synergy effect; therefore, the activating compound may be secreted. Taken together, our results point to complex, supportive interactions between members of the plant microbiome.

## Materials and methods

### Media

Microbial media used in this study included a modified nitrogen-limited combined carbon medium, NL-CCM [18], containing, per liter, sucrose (5 g), mannitol (5 g), sodium lactate (ml, 60%, v/v, 0.5 ml), monobasic potassium phosphate (0.8 g), dibasic potassium phosphate (0.2 g), sodium chloride (0.1), sodium molybdate (25 mg), and ferric sodium salt (28 mg). The pH was adjusted to 6.7, and, if for agar plates, Phytoblend agar (Phytotechnologies Inc) (15 g) was added prior to autoclaving. Following autoclaving, filter-sterilized yeast extract (100 mg) and autoclaved magnesium sulfate heptahydrate (200 mg) and calcium chloride (60 mg) were added. Other media included a nitrogen-free version of this medium lacking the yeast extract that we refer to as NF-CCM and the rich medium, MG/L [19]. Plant samples were processed in a nitrogen-free medium (NFM) [16].

### Plant extraction and initial screening using the acetylene reduction assay

Branches from a variety of unidentified lava field plants near Kona, Hawaii (May 2019), and roots from *Po. trichocarpa* in the riparian zone of the Skykomish River, Washington State, USA (August 2021), were collected. The plant samples were surface-sterilized by washing with a bleach detergent solution (10% commercial bleach for a final concentration of 0.6% active ingredient) for 5–10 min, followed by several rinses in sterile water. Material was ground in sterile mortars with pestles and extracted either in 40 ml sterile water (Hawaiian plant samples) or 25 ml NFM (poplar roots). One hundred microliters of the Hawaiian plant extracts were spread onto NF-CCM agar plates. Colonies, here meaning individual areas of growth, observed on the plates were restreaked onto NF-CCM three times to achieve isolation. Colonies were collected and suspended in NLCCM and tested by the acetylene reduction assay (ARA) as described below. The colony with the highest activity was then streaked onto rich media (MG/L) revealing synergistic strains living within the colony. The poplar root extracts were directly tested. A strongly active sample was extracted from the ARA vial and plated onto NF-CCM. Areas of growth were then streaked for isolation on both NF-CCM and MG/L agar plates.

### Isolation and cultivation of bacterial strains

Colonies that demonstrated nitrogenase activity from the acetylene reduction assay were streaked onto MGL and NF-CCM to isolate individual strains. Multiple passages of collecting colonies and streaking were required to separate strains for pure isolation. Cell suspensions of streak-purified strains were stored in glycerol at −80°C. Preliminary identification was accomplished through colony polymerase chain reaction (PCR) using primers (8F and 1492R) that amplified 1.5Kb of the 16S rRNA gene. Sequencing of the PCR products was performed by Azenta Life Sciences (Seattle) and analyzed using National Center for Biotechnology Information (NCBI) BLASTN.

### Construction of a nitrogenase mutant control strain

An 850 bp fragment of the nitrogenase subunit gene, *nifH*, was cloned from *Rahnella aceris* strain WP5 into pGEM-T-Easy (Promega). *In vitro* transposon mutagenesis was performed using the EZ-Tn5 < KAN-2 > Insertion Kit according to the manufacturer’s instructions (epicenter). A resulting plasmid with an insertion site mapping by restriction analysis to near the center of the *nifH* fragment was digested with EcoRI, yielding a 2Kb fragment (1.2Kb Tn5 plus 0.8Kb nifH). Counter-selectable pEXG2 (a kind gift of Joseph Mougous lab, UW Microbiology Department) was digested with EcoRI and ligated with the 2Kb fragment. The resulting plasmid was introduced into WP5 via conjugation with helper strain HB101 (pRK2013). Transconjugants were tested by PCR with b1 nifH primers [20] for the presence of the disrupted *nifH* product (1.6Kb) and the absence of the wild-type product (0.4Kb). The identity of the transconjugant was verified by PCR (8F and 1492R) and sequencing the 16S rRNA gene.

### Acetylene reduction assays

All bacteria were grown on nutrient-rich (MGL) agar plates at 30°C for 2–5 days. Nitrogen-fixing strains were also grown on NL-CCM agar plates at 30°C for 3–7 days. Bacteria were suspended in NFM, incorporating cells of nitrogen fixers grown on NL-CCM and MGL. The cells of each strain were then diluted to an optical density (OD_600_) of 0.4 unless noted in figure legends. The total volume of tested suspensions was 150 μl. When mixing isolated strains, the diluted suspensions were combined in equal parts (75 μl) in 17.5 ml amber vials (Millipore Sigma) that contained 6 ml of NL-CCM agar. For the heat-killed samples, the diluted suspensions were incubated at 95°C for 30 min on a heat block. Vials were sealed with septa caps (PTFE Silicone) and dosed with 100 μl of acetylene (99.6%) for a final concentration of 1%. Following 3 days of incubation at 30°C, 5 ml headspace was transferred to autosampler vials (Thermo-Fisher) and analyzed on a gas chromatograph equipped with a flame ionization detector (Trace GC Ultra, Thermo Scientific, Waltham, MA). A standard curve of ethylene concentrations was generated and used to calculate ethylene production. All gases (Ultra Zero compressed air, nitrogen, acetylene, and ethylene) were obtained from Linde Gas except for the hydrogen gas that was obtained from a generator (Domnick Hunter 20H-MD).

### Separation by 0.2 μm filter during acetylene reduction assay

The preparation of bacteria was consistent with that of the acetylene reduction assay above; however, 4 ml of diazotroph suspension was placed in a 40 ml amber vial. The funnel chamber and lid were removed from a Corning Costar SpinX tube. The funnel was filled with 300 μl of a synergistic partner in an OD_600_ of 0.8 suspension. Using a water-filled vial as a spacer, the funnel was then situated such that only the 0.22 μm membrane was submerged in the suspension of diazotroph (Fig. S5). The headspace was then dosed with 250 μl of acetylene to achieve a ~1% final concentration. Vials were then incubated and tested as above.

### Construction of constitutive fluorescent diazotroph strains

For *Rahnella* sp. WP5, the promoterless Green Fluorescent Protein (GFP) was cloned into the plasmid pUC18-miniTn7T2-Gm-mCherry [21] in place of the existing mCherry using *in vivo* DNA assembly and cloning as described [22]. All DNA fragments, including the synthesized fragments, were PCR-amplified using high-fidelity DNA polymerases PrimeSTAR Max (2× Master Mix, Takara Bio). Primers and templates are listed in Table S9. When applicable, PCR products were subjected to DpnI digest (New England Biolabs; CutSmart buffer was added to a final concentration of 1× for all digests) for ~2 h at 37°C. The PCR Clean-Up kit (Qiagen) was used for PCR product purification. Plasmid assembly was done by transforming DNA fragments into *Escherichia coli* DH5α (Subcloning Efficiency DH5α Competent Cells, Invitrogen). *Azospirillum* sp. 11RA and *Azorhizobium* sp. HT1-9 were both transformed with the plasmid pBHR-GFP-Km to induce constitutive fluorescence with GFP. All three strains were transformed via electroporation as described [23] (Electroporator 2510, Eppendorf) at 2500 V in 0.2 mm cuvettes and plated for selection on MGL agar plates with appropriate antibiotics.

### Growth curves

GFP-tagged diazotrophs and synergistic helpers were cocultured aerobically in an NL-CCM medium in 24-well plates (Corning) at 30°C and 100 rpm for 48 h. Each well contained one diazotroph and either no helper or one helper, both at a starting OD_600_ of 0.1. GFP (489/520) and OD_600_ measurements were taken at 0, 24, and 48 h in a BioTek Synergy H1 Multimode Reader.

#### Statistical analysis

Data are reported as the means ± 95% confidence intervals (*n* = 3–9). Statistical analyses were performed using R (v4.3.1) in RStudio (v2023.12.0). Descriptive statistics were calculated using the rstatix package (v0.8.2) [24]. Single-factor Analysis of Variance (ANOVAs) with Type III sum of squares (SS) to account for unbalanced designs, or independent *t*-tests, were used to test for differences among groups (α = 0.05). ANOVAs were performed using the Anova() function from the car package (v3.1.2) [25]. *Post hoc* pairwise comparisons were conducted using estimated marginal means with Bonferroni adjustment (α = 0.05), and Cohen’s *d* effect sizes were calculated using the emmeans package (v1.10.0, 26). Full R packages and dependencies are available in the R scripts deposited in the figshare repository (see Data Availability).

#### Genomic sequencing and analysis

Genomic DNA was purified using standard protocols with phenol/chloroform extraction and ethanol precipitation as described [26]. The whole genome sequencing was performed for 12 endophyte strains (Table S1). The library construction, amplification, and sequencing of the Hawaiian strains *Azorhizobium* HT1-9, *Brevundimonas* HT1-5, *Rhizobium* HT1-8, *Rhizobium* HT1-10, and *Sphingobium* HT1-2 were completed using Illumina MiSeq technology by Genewiz (Azenta Life Sciences, https://www.genewiz.com). The genome of the Hawaiian isolate, HT1-6, was not sequenced, but a megablast analysis of the 16S rRNA gene placed it in the *Azorhizobium* genus (Supplementary Files 2a and b, Figshare). The Washington strains *Azospirillum* 11R-A, *Fondihabitans* 4ASC-45, *Herbiconiux* 11R-BC, *Sphingobium* 11R-BB, and *Sphingomonas* 4RDLI-65, were sequenced by Novagene Co. Ltd (https://www.novogene.com) using an Illumina NovaSeq 6000 platform. *Ra. aceris* WP5 was sequenced by the DOE Joint Genome Institute (https://genome.jgi,doe.gov) using an Illumina HiSeq 2000 platform. Unicycler v0.5.0 [27] was used to assembly a hybrid draft genome of *Sphingobium* WW5 from long-read (Oxford Nanopore MinION) and short-read sequences (Illumina MiSeq) by Intrinsyx Bio (https://intrinsyxbio.com). The raw sequence reads of the other strains were uploaded to the Bacterial and Viral Bioinformatics Center (BV-BRC, https://www.bv-brc.org) for assembly, annotation, and systems/pathways analysis via the Comprehensive Genome Analysis pipeline [28]. The assembly method used was SPAdes v3.13.0 (Bankevich2012), the draft genomes were annotated via RAST-tk v1.073 [29], and completeness and contamination were assessed using CheckM [30]. The genomic sequence data for WP5 was downloaded on 3 March 2023 from the JGI IMG/MER database [31] and uploaded to BV-BRC, along with WW5, for annotation and systems/pathways analysis in order to maintain consistency with the other strains. The draft genome assemblies were submitted to the Type (Strain) Genome Server (TYGS) for whole-genome-based taxonomic classification and phylogenetic analysis. The strains with digital DNA:DNA hybridization (dDDH) values ≥70% were assigned to the same species as the type-strain match, while those with dDDH values <70% (formula *d_4_*) were classified as potentially novel species [32].

## Results

### Proof of principal from Site 1: non-diazotrophic strains co-isolate with and synergistically enhance nitrogenase activity of diazotrophs

**Figure 1:**
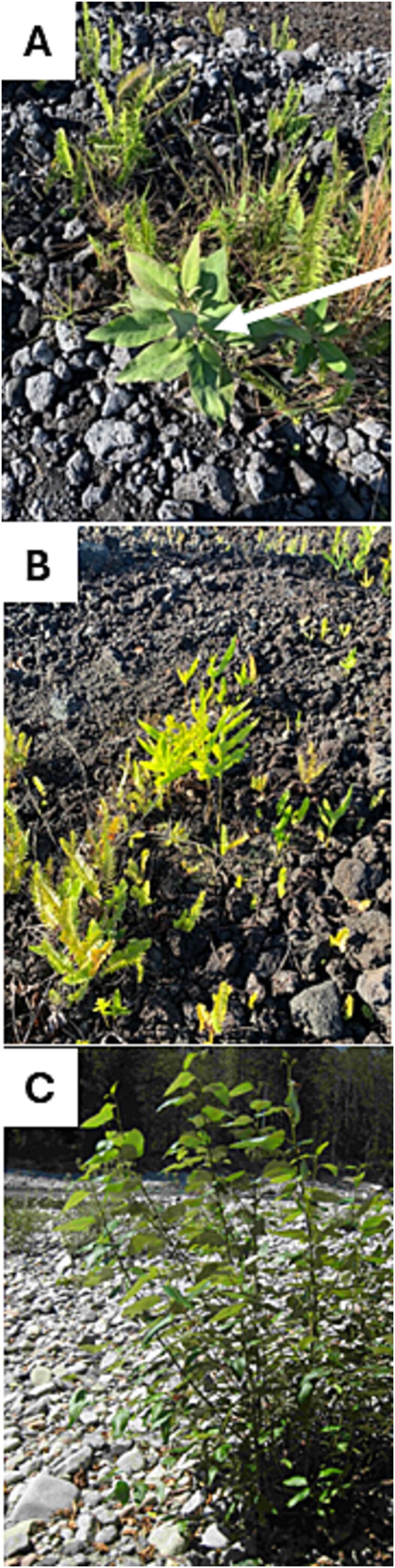
Tissues from a variety of plants growing in nutrient-limited sites were selected to screen for endophytic nitrogen-fixing bacteria. (A, B) Plants growing in a Hawaiian lava field near Kona, Hawaii, USA. The white arrow in (A) is representative of plant HT1. (C) *Po. trichocarpa* at the Skykomish River in Washington State, USA.

The extracts from a variety of plants including ferns, succulents, and other plants that were thriving on a Hawaiian lava field (Fig. 1) were plated onto nitrogen-free medium (NF-CCM) and yielded areas of growth that were tested for nitrogenase activity using the ARA. The plant extract sample HT1 (from a plant tentatively identified as *Pluchea carolinensis*) displayed the strongest activity (Fig. S1) and, when streak-purified onto a rich medium, revealed that what had appeared to be a single colony on NFM was actually 10 morphologically distinct strains (Fig. S2). 16S rRNA gene sequencing identified four strains to be among known N-fixing genera, two *Azorhizobium* sp. (HT1-6 and HT1-9), and two *Rhizobium* sp. (HT1-8 and HT1-10). Testing of individual strains by ARA indicated that only HT1-6 and HT1-9 had nitrogenase activity (data not shown). However, the activity of the individual diazotrophic strains was observed to be far less than that of the original HT1 sample (Fig. S3).

**Figure 2:**
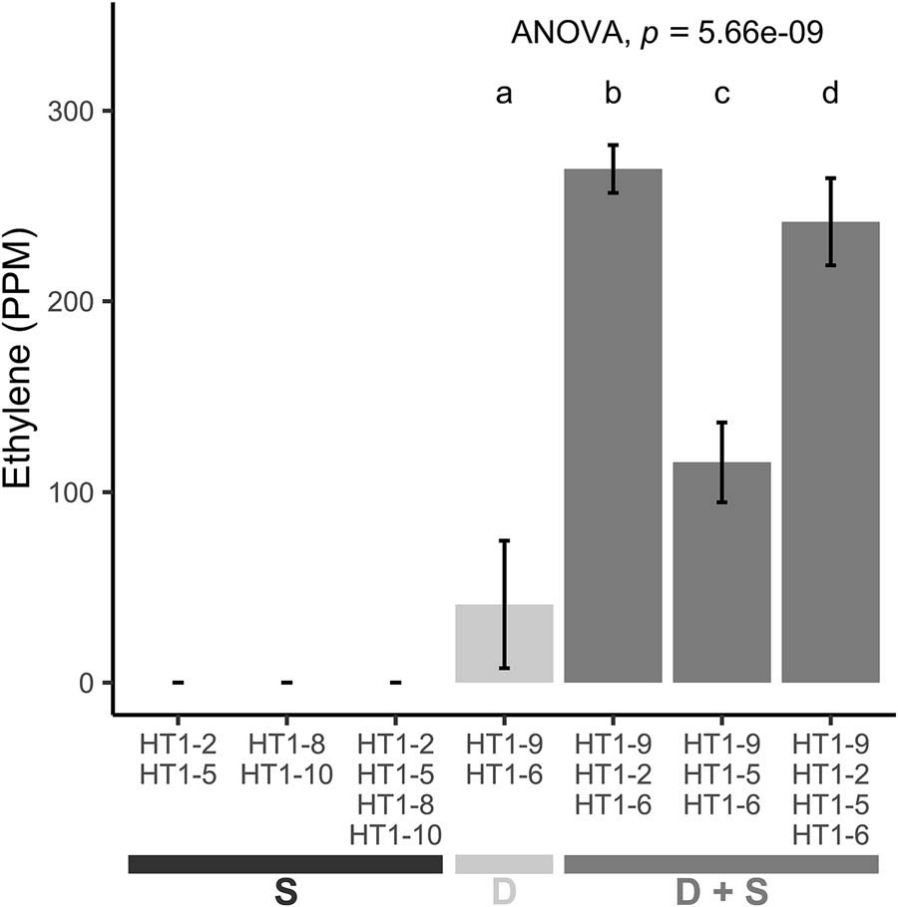
Bacterial cocultures modulate nitrogenase activity in the diazotrophs *Azorhizobium* HT1-6 and HT1-9. Coculturing *Azorhizobium* spp. HT1-6 and HT1-9 with *Sphingobium* sp. HT1-2 or *Brevundimonas* HT1-5 enhanced nitrogenase activity (seen as ethylene from the reduction of acetylene) compared to HT1-6 and HT1-9 alone (*F* [3, 8] = 382.131, *P* < < .001), with HT1-2 exhibiting a greater effect than HT1-5 (Cohen’s *d* = 24.006 vs. 7.831). Combining HT1-2 and HT1-5 moderately decreased nitrogenase activity compared to HT1-2 alone (Bonferroni, *P* = .003, Cohen’s *d* = 2.914). No acetylene reduction was detected in the groups without HT1-6 and HT1-9, including the two *Rhizobium* strains, HT1-8 and HT1-10; these data were excluded from the statistical analysis. Data are mean ± 95% CI (*n* = 3); one-way ANOVA. Different letters indicate significant differences (*P* < .001). Legend key: D, diazotroph; S, synergist; D + S, diazotroph and synergist.

To test if the non-diazotrophic strains enhanced the nitrogenase activity of the *Azorhizobium* isolates, we tested each nonfixing strain for synergistic properties when mixed with cocultures of the diazotrophic strains, HT1-9 and HT1-6 (Fig. 2, Table S2). Strains HT1-2 (*Sphingomonas* sp.) and HT1-5 (*Brevundimonas* sp.), alone or in combination, increased nitrogenase activity in the diazotrophs (*F* [3, 8] = 382.1, *P* < .001). The synergistic effect of HT1-2 on the nitrogenase activity of the diazotrophs was much greater than that of HT1-5 (*P* < .001), and coculturing HT1-2 and HT1-5 together resulted in a moderate decrease in activity compared to HT1-2 alone (*P* = .044). This suggests that their mechanisms for promoting nitrogenase activity are not complimentary. Strain HT1-2, a yellow-pigmented *Sphingomonas* sp. strain, was therefore identified as a synergistic partner.

### Proof of principal from Site 2: synergistic enhancement of nitrogenase activity occurs in additional nutrient-limited environments

Our previous work demonstrated that nitrogen fixation occurs in black cottonwood (*Po. trichocarpa*) growing in a cobble-dominated, nutrient-limited riverbank of the Snoqualmie River in Washington State [33]. To investigate whether the synergistic phenomenon observed with the Hawaiian endophytes is also observed with diazotrophs from other nutrient-limited environments, we collected samples from poplar trees growing in similar conditions along the Skykomish River, further north in Washington State (Fig. 1). An ARA-positive extract from the poplar roots was plated on NFM, and the dominant diazotroph was identified as *Azospirillum* sp. strain 11R-A. As observed with the Hawaiian samples, two nondiazotrophic, yellow-pigmented strains co-isolated with 11R-A, designated as *Sphingobium* sp. 11R-BB and *Herbiconiux* sp. 11R-BC, both of which increased 11R-A’s nitrogenase activity in ARA experiments (data not shown).

We further tested the generality of synergism with diazotrophs by screening our library of nondiazotrophic strains that we previously isolated from the Snoqualmie River site, using a mixed diazotroph culture of *Ra. aceris* WP5 (a low-activity strain) [34] and *Burkholderia vietnamiensis* WPB (a high-activity diazotrophic strain) [16, 35]. *Sphingobium* sp. WW5 [34], originally isolated from wild willow (*Salix* sp.), was identified as a strong synergist, amplifying the diazotroph mix’s activity over 50-fold (Fig. S4). Other strains tested had weak, zero, or even negative impacts on nitrogenase activity. Therefore, the diazotrophic synergy effect is not a universal trait.

### Genomic characterization suggests a high potential for novel diversity in endophytic bacteria

We characterized the genome sequences of twelve bacterial endophytes isolated from plants in Washington State (*Po. trichocarpa*, *Po. tremuloides*, *Salix sitchensis*, and *Cornus sericea*) and Hawaii (*Pl. carolinensis*) (Table S1). The endophytes represent three classes (Alphaproteobacteria, Gammaproteobacteria, and Actinobacteria) and eight genera commonly associated with plants: *Azorhizobium*, *Azospirillum*, *Brevundimonas*, *Frondihabitans*, *Herbiconiux*, *Rahnella*, *Rhizobium*, and *Sphingobium*. The draft assemblies were good quality, with CheckM2-completeness of at least 98% and CheckM2-contamination of no more than 1.8%, and the genomes ranged in size from 3.10 Mb (*Brevundimonas* HT1-5) to 7.87 Mb (*Azospirillum* 11R-A), and guanine cytosine (GC) content varied from 52.15% (*Rahnella* WP5) to 70.30% (*Herbiconiux* 11R-BC).

Taxonomic classification analysis showed that 10 of the 12 strains (83.3%) are potentially novel species. The TYGS pipeline assesses taxonomic relationships using genome-scale phylogenetics and digital DNA–DNA hybridization (dDDH), with species and subspecies boundaries determined using established dDDH thresholds of 70% and 79%, respectively [32] (the full TYGS reports can be found in the Figshare repository). The two strains, *Ra. aceris* WP5 and *Rhizobium wenxiniae* HT1-8, were assigned to species based on their high similarity to the type strains *Ra. aceris* SAP-19 (90.3%) and *Rh. wenxiniae* DSM 100734 (78.6%), respectively. The remaining strains were below the species delineation threshold (70%). Four strains had very low genomic similarity to their closest type strains: *Frondihabitans* 4ASC-45 (21.4% with *Frondihabitans australicus* DSM 17894), *Herbiconiux* 11R-BC (24.5% with *Herbiconiux ginsengi* CGMCC 4.3491), *Rhizobium* HT1-10 (26.2% with *Rh. tumorigenes* 1078), *Azorhizobium* HT1-9 (37.4% with *Azorhizobium oxalatiphilum* CCM7897), while the remaining strains had moderate genomic similarity to the closest type strain: *Brevundimonas* HT1-5 (60.2% with *Brevundimonas vesicularis* NBRC 12165), *Azospirillum* 11R-A (65.2% with *A. palustre* B2), and the three *Sphingobium* strains, WW5 (66.3%), 11R-BB (66.2%), and HT1-2 (68.1%), with the type strain *Sphingobium yanoikuyae* ATCC 51230.

The three *Sphingobium* strains, WW5, 11R-BB, and HT1-2, were more genomically similar to each other, particularly WW5 (isolated from *S. sitchensis* stem) and 11R-BB (isolated from *Po. trichocarpa* root), than with *S. yanoikuyae* ATCC 51230. A comparative TYGS analysis of the three *Sphingobium* strains indicates that WW5 and 11R-BB form a single subspecies (*d4* = 100.00), while both showed high genomic similarity to HT1-2 (*d4* ≈ 70.0–70.1). Despite their close genomic relationships, the three strains displayed distinct effects on nitrogenase activity when interacting with diazotrophic strains. A combined phenotypic and phylogenetic analysis supports the hypothesis that WW5 and 11R-BB represent one novel species (proposed as *S. salicis*), distinct from HT1-2, which is proposed as a second novel species (manuscript in preparation).

**Figure 3:**
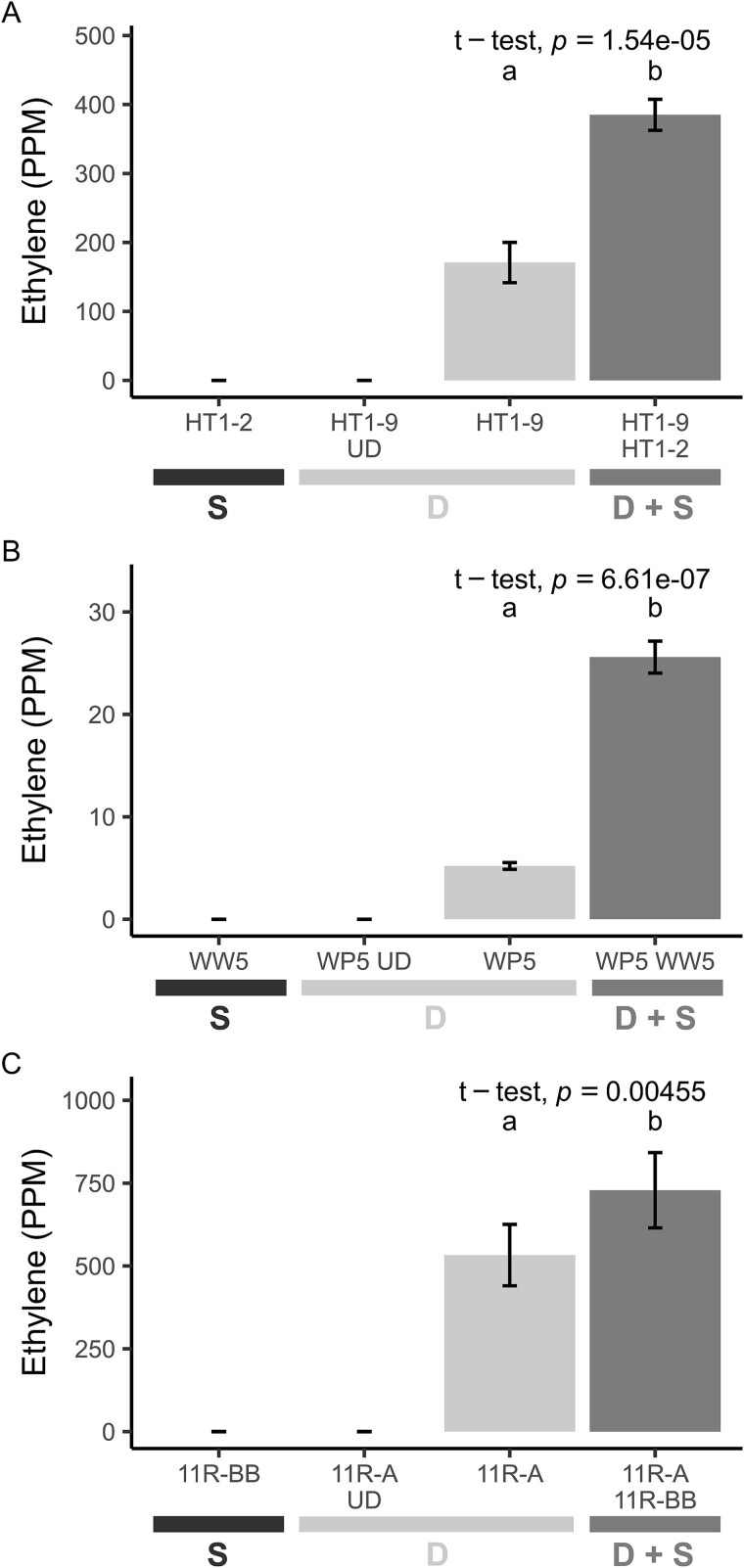
*Sphingobium* strains enhance nitrogenase activity in diazotrophic cocultures. Nitrogenase activity was measured in cocultures of diazotrophs (*Azorhizobium* sp. HT1-9, *Ra. aceris* WP5, *Azospirillum* sp. 11R-A) with the *Sphingobium* synergists (HT1-2, WW5, and 11R-BB, respectively). (A) Nitrogenase activity was 2.3-fold higher in the HT1-9/HT1-2 coculture compared to HT1-9 alone (*t* [4] = −24.935, *P* < < .001), (B) 4.9-fold higher in the WP5/WW5 coculture compared to WP5 alone (*t* [4] = −54.861, *P* < < .001), and (C) 1.4-fold higher in the 11R-A/11R-BB coculture compared to 11R-A alone (*t* [4] = −5.744, *P* = .005). No acetylene reduction was detected in the undosed controls or synergist-only groups; these data were excluded from the statistical analysis. Data are mean ± 95% CI (*n* = 3); independent *t*-tests. Different letters indicate significant differences (*P* < .01). Legend key: D, diazotroph; S, synergist; D + S = diazotroph and synergist.

**Figure 4:**
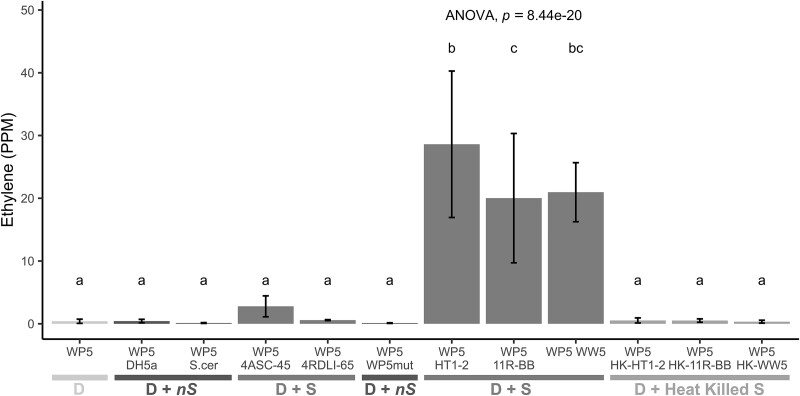
Significantly increased nitrogenase activity of *Ra. aceris* WP5 when cocultured with any of the synergy strains. The nitrogenase activity of WP5 was measured alone or in coculture with live or heat-killed bacterial or yeast strains. Activity increased only with the live *Sphingobium* cells (*P* < .001), with HT1-2 having the strongest effect (Cohen’s *d* = 7.413); no effect on WP5’s nitrogenase activity was observed with the heat-killed cells. Nitrogenase activity was slightly increased with *Frondihabitans* 4ASC-45 (*P* < .05, Cohen’s *d* = 0.700) but not with *Sphingomonas* 4RDLI-65. *Saccharomyces cerevisiae*, *E. coli* DH5α, and the *nifH* mutant WP5*mut* had no effect on the nitrogenase activity of WP5. Data are mean ± 95% CI (*n* = 5); one-way ANOVA, different letters indicate significant differences (Tukey’s HSD, *P* < .001). Legend key: D, diazotroph; S, synergist; D + S, diazotroph and synergist; D + nS, diazotroph + nonSynergist; D + heat-killed S, diazotroph and heat-killed synergist.

As confirmation of synergism, we performed ARA on the *Spingobium* strains, HT1-2, WW5, and 11R-BB, and the diazotrophs, HT1-9, WP5, and 11R-A, respectively, along with undosed (no acetylene) controls (Fig. 3**,**Table S3). The nitrogenase activity of all three diazotrophs increased in the presence of their synergist partner compared to when cultured alone. The nitrogenase activity in the low-activity diazotrophic strain WP5 increased 4.9-fold in the presence of synergist WW5 (*P* < .001), while the higher-activity diazotrophic strains HT1-9 and 11R-A increased by 2.3- and 1.4-fold when cultured with the synergist strains HT1-2 and 11R-BB, respectively (*P* < .01). WP5, the low-activity diazotroph, exhibited a particularly strong response to coculturing with each of the *Spingobium* synergists (Fig. 4), revealing that the synergistic strains can partner with multiple diazotrophic species. To test the specificity of this response, we included trials with two nonsynergistic endophyte strains (*Frondihabitans* sp. 4ASC-45 and *Sphingomonas* sp. 4RDLI-65), *E. coli* DH5α, *Saccharomyces cerevisiae*, and a WP5*mut* a *nifH* mutant. Nitrogenase activity did not increase with *E. coli*, *S. cerevisiae*, WP5*mut*, or 4RDLI-65 (*P* > .05). While 4ASC-45 increased WP5 N-fixing activity by nearly 7-fold, the magnitude of this effect was insignificant compared to the *Sphingobium* synergists. These results, along with those from synergist *Herbiconiux* sp. 11R-BC, suggest that this phenomenon may extend to other bacterial taxa.

**Figure 5:**
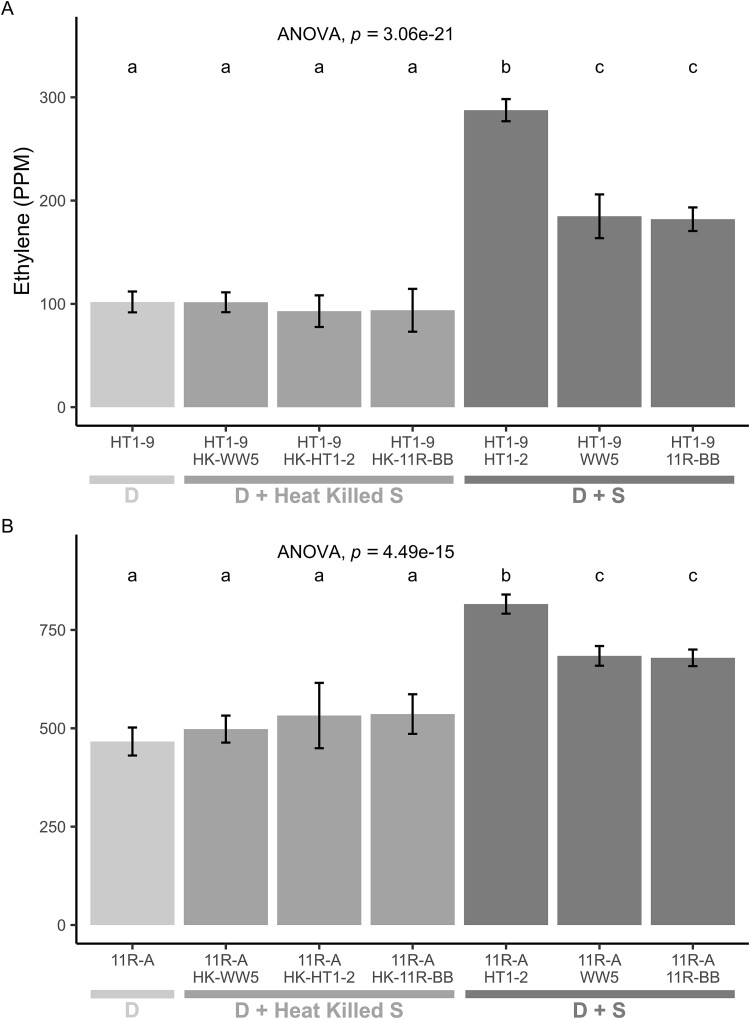
Synergistic enhancement of nitrogenase activity by *Sphingobium* strains requires live cells. Nitrogenase activity was measured in cocultures of diazotrophs (A) *Azorhizobium* sp. HT1-9 or (B) *Azospirillum* sp. 11R-A with live or heat-killed *Sphingobium* strains HT1-2, WW5, or 11R-BB. For HT1-9, coculture with live HT1-2, WW5, and 11R-BB increased nitrogenase activity by 2.8-fold, 1.8-fold, and 1.7-fold, respectively, compared to HT1-9 alone (F6,28 = 186.505, p < < 0.001). For 11R-A, live HT1–2, WW5 and 11R-BB increased nitrogenase activity by 1.7-fold, 1.5-fold, and 1.5-fold, respectively (*F* [6, 28] = 64.248, *P* < < 0.001). Heat-killed synergists had no effect on nitrogenase activity in either diazotroph (Bonferroni, *P* > .05). Data are mean ± SE (*n* = 5), one-way ANOVA. Different letters indicate significant differences (*P* < .05). Legend key: D, diazotroph; S, synergist, D + S, diazotroph and synergist; D + heat-killed S, diazotroph and heat-killed synergist.

Having established that only the identified synergists were able to strongly amplify the activity of WP5, we then tested the three synergists with the Hawaiian diazotrophic strain HT1-9 and the riparian diazotrophic strain 11R-A. The Hawaiian lava field endophyte synergist strain, HT1-2, was the most effective synergy strain, amplifying the nitrogenase activities of not only the Hawaiian diazotroph, *Azorhizobium* sp. strain HT1-9 but also that of the *Azospirillum* strain 11R-A and of the *Ra. aceris* strain WP5 (Figs 4 and 5). WW5 and 11R-BB had comparable effects to each other.

### Characterization of mechanisms of the synergy effect

To determine if active metabolism is required for the synergistic effect, we measured the nitrogenase activity of WP5, 11R-A, and HT1-9 with either live or heat-killed cells of the synergist *Sphingobium* strains HT1-2, WW5, and 11R-BB and found that activity increased only in the cultures with live cells, regardless of the specific diazotroph–synergist pair (*P* < .001 vs. heat-killed controls) (Figs 4 and 5, Tables S4 and S5). HT1-2 consistently had the largest effect on nitrogenase activity among the three synergists, while WW5 and 11R-BB had comparable effects.

Since heat-killed helper strains and nonendophytes did not affect nitrogenase activity, the synergy mechanism does not appear to be simply nutritional. Measuring the growth of cocultures of diazotrophs with helpers after 48 h showed no growth enhancements for the diazotroph, offering further support for nutrition not being the driver of increased activity (Table S10). If a quorum sensing (QS) signal [36] that could be provided by strains with the same QS molecule as the diazotroph is the amplifying mechanism, then we reasoned that a higher concentration of the diazotroph itself would enhance nitrogenase activity. This effect would be difficult to separate from higher activity simply from having more diazotroph cells so we tested the addition of a WP5 nitrogenase (*nifH*) mutant. This strain had no enhancing activity (Fig. 4), ruling out this type of QS mechanism.

**Figure 6:**
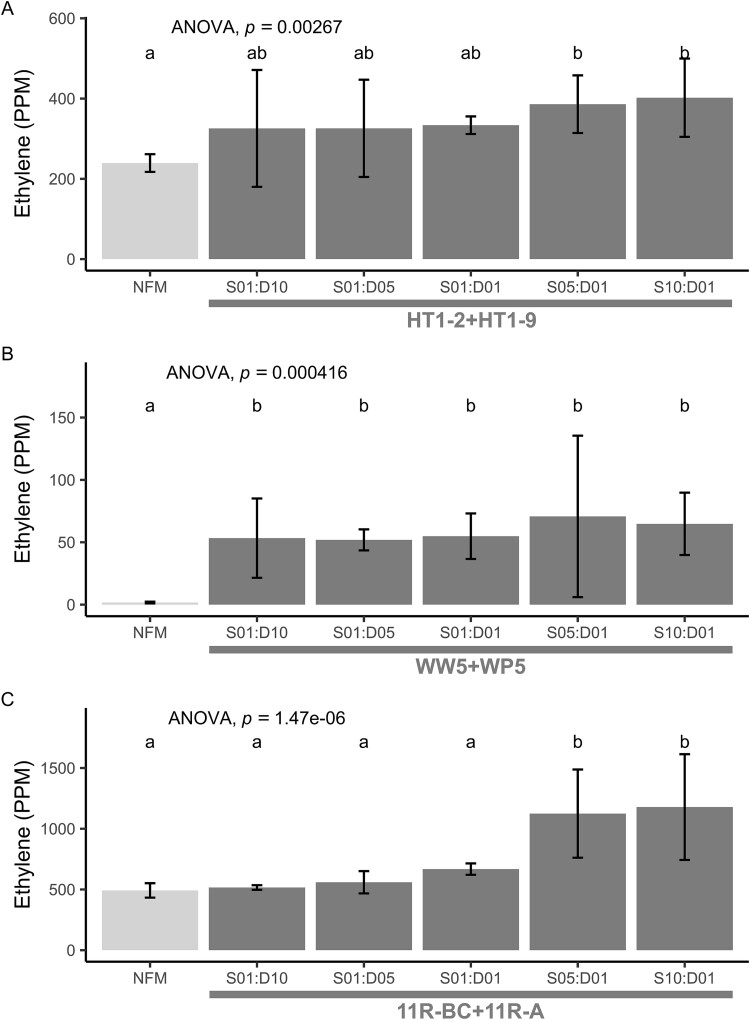
Effect of synergist to diazotroph ratio on nitrogenase activity in cocultures. (A) *Azorhizobium* sp. HT1-9 and *Sphingobium* sp. HT1-2, (B) *Ra. aceris* sp. WP5 and *Sphingobium* sp. WW5, and (C) *Azospirillum* sp. 11RA and *Herbiconiux* sp. 11R-BC. Nitrogenase activity was measured at various synergist to diazotroph ratios (S01:D10, S01:D05, S01:D01, S05:D01, and S10:D01) in NFMs, with S01:D01 representing a starting OD_600_ of 0.5. The presence of the synergist enhanced nitrogenase activity compared to the NFM control in all experiments. For HT1-9/HT1-2 (A) and WP5/WW5 (B), higher synergist proportions (S05:D01 and S10:D01) showed greater activity than the NFM control (Bonferroni, *P* < .01). For 11R-A/11R-BC (C), increasing the synergist proportion while keeping the diazotroph constant (D01) enhanced activity (Bonferroni, *P* < .01). Statistically varying the diazotroph proportion with a constant synergist (S01) had minimal impact on activity in the HT1-9/HT1-2 and WP5/WW5 pairs but increased activity in the 11R-A/11R-BC. Data are mean ± 95% CI (*n* = 3), one-way ANOVA. Different letters indicate significant differences (*P* < .05).

We therefore examined the influence of relative concentration on the synergy phenotype by pairing the diazotrophs with the synergist *Sphingobium* strains at different synergist-to-diazotroph ratios and measuring the resulting nitrogenase activity. We found that the effect of varying ratios was specific to the synergist–diazotroph pairing (Fig. 6, Table S6). For HT1-9, nitrogenase activity increased in the presence of the synergist HT1-2 (*F* [5, 12] = 7.1, *P* = .003), but only significantly at the 5:1 and 10:1 synergist-to-diazotroph ratios (*P* < .01) (Fig. 6A). Diazotroph WP5 did not follow this pattern. The mere presence of the synergist WW5 increased nitrogenase activity (*F* [5, 12] = 10.8, *P* < .001), but further increases in the synergist-to-diazotroph ratio had no additional effect on nitrogenase activity (Fig. 6B). Unique among the diazotrophs, *Azospirillum* strain 11R-A was strongly influenced by the higher synergist-to-diazotroph ratios (*F* [5, 12] = 32.27, *P* < .001) (Fig. 6C). A significant increase was observed at the highest 11R-BC-to-11R-A ratios of 5:1 and 10:1 (*P* < .001), nearly doubling the activity at a ratio of 10:1. Of the two helper strains that co-isolated with 11R-A, strain 11R-BB was determined to be nearly identical to strain WW5; therefore, only 11R-BC was used as the partner for 11R-A.

**Figure 7:**
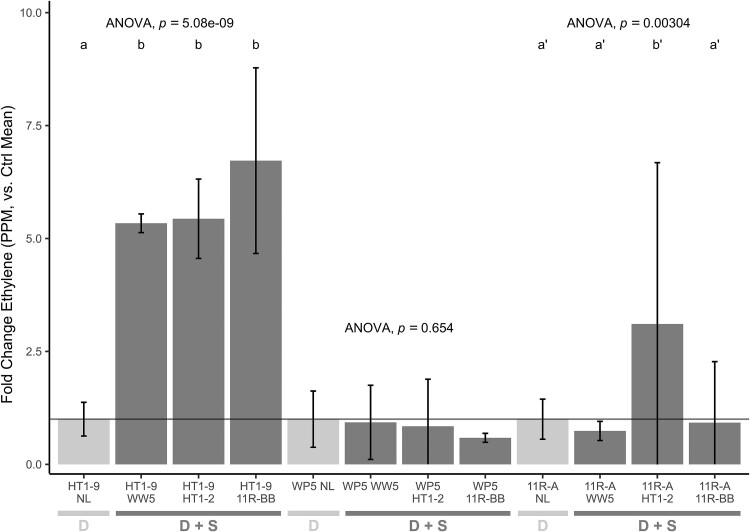
Normalized effect of synergists on nitrogenase activity in 0.2 um filter-separated cultures. Nitrogenase activity was measured in a nitrogen-limited medium (NL) and normalized against the mean ppm of the NL group for each diazotroph (represented by the black line at 1.0). For HT1-9, all three *Sphingobium* synergists (WW5, HT1-2, and 11R-BB) increased normalized nitrogenase activity compared to the NL control (Bonferroni, *P* < < .001), while none affected activity in WP5 (Bonferroni, *P* > .05). HT1-2 increased normalized activity in 11R-A compared to NL and the other synergists (Bonferroni, *P* < .01), and WW5 had no effect (Bonferroni, *P* > .05). Data are presented as mean ± 95% CI (for HT1-9 and WW5 in NL *n* = 6, and with synergists, *n* = 3, for 11R-A in NL, *n* = 9, and with synergists, *n* = 3), one-way ANOVA (Type III SS). Different letters indicate significant differences (*P* < .05).

To investigate if the synergy effect required physical contact or is mediated by diffusible molecules, an experimental set-up was designed to compartmentalize the two strains but still allow for metabolic exchange (Fig. S5). The ARA results indicated that compartmentalization of the synergy strains, WW5 and HT1-2, did not prevent the enhancement of HT1-9 nitrogenase activity (*F* [3, 14] = 79.2, *P* < .001) (Fig. 7, Table S7), suggesting that the effect is mediated by a diffusible molecule. However, compartmentalization did prevent the synergistic effect with diazotroph WP5 (*F* [3, 11] = 0.56, *P* = .654). The mechanisms at play for diazotroph 11R-A may be even more complex and depend on the synergistic partner (*F* [3, 14] = 7.55, *P* = .003), with the synergy effect being blocked if WW5 or 11R-BB were compartmentalized (*P* > .05) but not if HT1-2 was compartmentalized (*P* = .004). Overall, the physical separation experiments indicated that there are multiple mechanisms underlying the enhancement of nitrogenase activity by the synergists.

**Figure 8:**
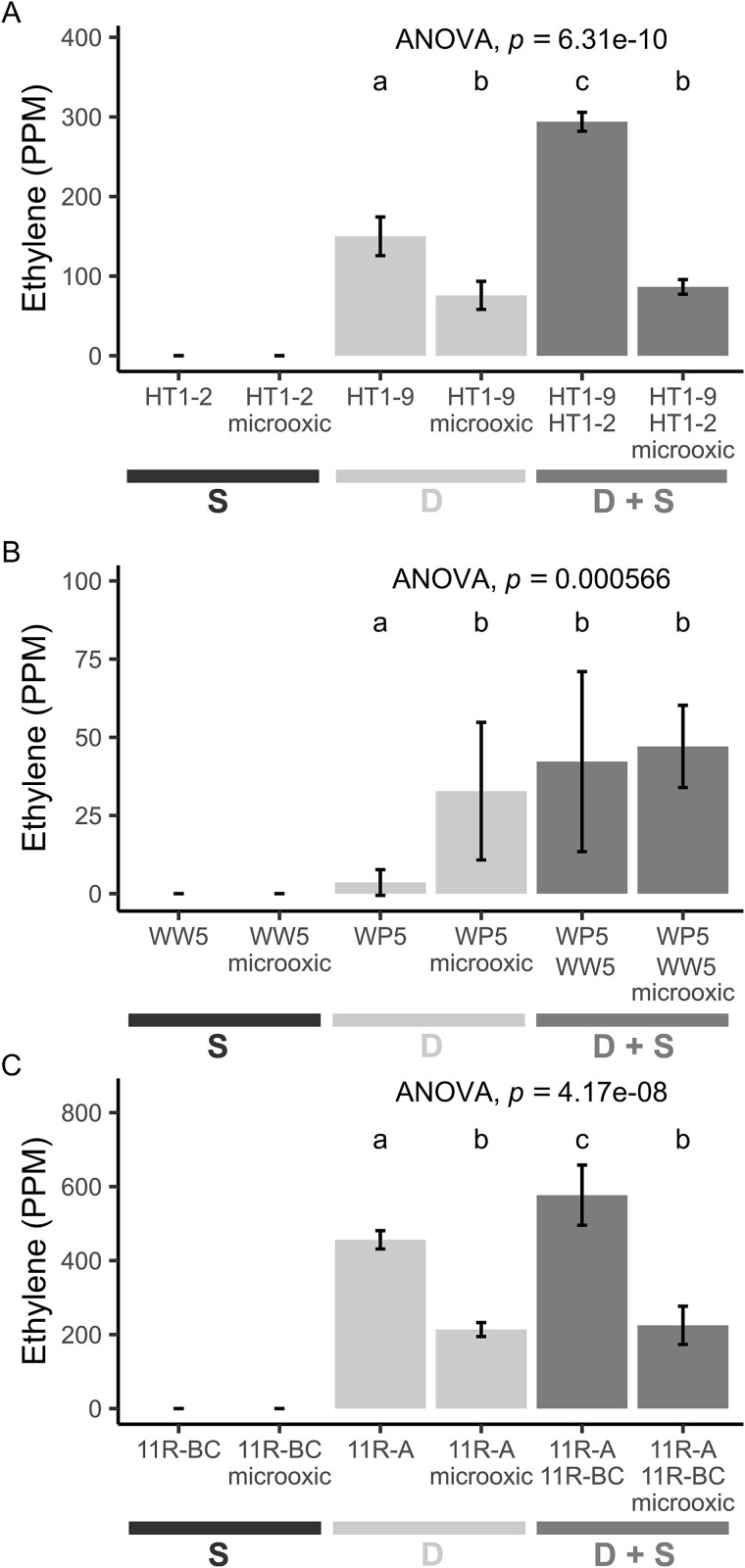
Effect of a microoxic environment on nitrogenase activity. Nitrogenase activity was measured under ambient and microoxic conditions for (A) *Azorhizobium* sp. HT1-9, B) *Ra. aceris* WP5, and (C) *Azospirillum* sp. 11R-A, alone and in coculture with their respective synergists (*Sphingobium* sp. HT1-2, *Sphingobium* sp. WW5, and *Sphingobium* sp. 11R-BB). For HT1-9 (A) and 11R-A (C), microoxic conditions reduced activity both when grown alone and in coculture with their synergists (Bonferroni, *P* < < .001). For WP5 (B), microoxic conditions increased nitrogenase activity when grown alone (Bonferroni, *P* < .01) but had no effect when cocultured with its synergist WW5 (Bonferroni, *P* > .05). Data are mean ± 95% CI (*n* = 3), one-way ANOVA. Different letters indicate significant differences (*P* < .05).

For the case of diazotroph WP5 in particular, where physical interaction with the synergistic partner is required, the function of the nitrogenase amplifier may be to create a microoxic environment. To test this hypothesis, we measured the nitrogenase activity of the diazotrophs under microoxic conditions (1% oxygen) (Fig. 8, Table S8). The nitrogenase activity was improved in this microoxic environment only for strain WP5, while HT1-9 and 11R-A showed the highest activity in the ambient conditions, indicating that these two diazotrophs are aerobic strains. For HT1-9 and 11R-A, which did not require physical contact, the synergistic effect increased the nitrogenase activity only under ambient oxygen conditions (*P* < .001 for both), but not under microoxic conditions (*P* > .05), indicating that the function of the synergist is not to reduce oxygen. In contrast, the presence of the synergist and the microoxic conditions elicited a similar response in WP5’s nitrogenase activity (*F* [3, 8] = 18.7, *P* < .001) (Fig. 8B). Under ambient conditions, WW5 enhanced the activity of WP5 (*P* = .002), whereas, under microoxic conditions, there was no difference between the nitrogenase activity of WP5 with or without WW5 (*P* > .05). These data show that at least two mechanisms for the enhancement are at play, one via exported molecules and another by reduction of oxygen.

## Discussion

Dobereiner coined the term endophytic diazotroph in the 1990s, opening the possibility of tapping into nitrogen-fixing symbioses for nonleguminous plants [37]. Individual diazotrophic strains have been used as inocula, but since any reduction in the required chemical fertilizer would benefit the economics and environmental impacts of agriculture, the quest for strains that could potentially reduce or replace nitrogen fertilizer requirements has continued. More recently, our understanding of the role of microbiomes has developed into a conceptual understanding of the holobiont. Therefore, it is important to investigate interactions to better understand how diazotrophic strains can be optimized.

In this study, we described natural microbial partnerships that enhance nitrogenase activity. We found specific synergistic strains co-isolating with diazotrophic endophytic strains in environments as diverse as lava fields to rocky riparian zones, with the commonality being nutrient deficiencies. Through investigations to decipher the mechanisms for the synergistic effect, we found that it required living interactions but that multiple mechanisms are at play. For the diazotroph strain WP5, a filter barrier separating it from the helper strain prevented the nitrogenase activity enhancement (Fig. 7). WP5 was also the only diazotroph to be enhanced by a microoxic environment (Fig. 8B). This points toward oxygen depletion or protection, which is well understood for the functionality of the nitrogenase enzyme, as the mechanism of enhancement in WP5.

For diazotrophs HT1-9 and 11R-A, however, a microoxic environment was not sufficient to enhance nitrogenase activity. In fact, less activity was seen for the partnerships under microoxic environments (Fig. 8A and C). Since physical separation did not prevent diazotrophs HT1-9 and 11R-A from responding to the synergy strains, this result implies that an activating compound is secreted. Strain 11R-A may be more efficient at taking up this activating compound. In the experiment in which increasing ratios of the helper strain to diazotroph were used, HT1-9 responded linearly to increasing amounts of the helper strains, while 11RA’s response was more robust, supporting this hypothesis. Overall, our results indicate the complexity of diazotroph–helper interactions with varying abilities in the helper strain to export the inducing compound and in the diazotroph to import it. In current studies, we are using stable isotope probing for the identification of the nitrogenase activator.

*Sphingobium* species, of which there are over a hundred, are known to promote plant growth and increase stress tolerance [38]. Genomic analysis of strains HT1-2, 11R-BB, and WW5 indicated that they are novel species (manuscript is in preparation). This genus comprises strictly aerobic, gram-negative bacteria and is commonly found in plants, soil, and surface and aquifer water. *Herbioconium* sp. strain 11R-BC is unrelated to the other two helper strains. *Herbiconium* species are Actinomycetes in the Microbacteriaceae family, gram-positive, and not well described in the literature. Having such genetically diverse strains with the same nitrogenase-enhancing effect may aid in elucidating mechanisms based on genomic comparisons.

Most of the data for this study were generated through the acetylene reduction assay. This assay is commonly used as a proxy for nitrogen fixation capacity based on the ability of the nitrogenase enzyme to reduce the triple bond in acetylene to ethylene gas that is easily measured by gas chromatography [39]. The assay is highly sensitive, with a range of detection of ~10 000 times between minimum and maximum [39]. Ethylene concentration is quantified using a standard curve. In addition, the acetylene concentration remaining after the incubation period can serve as an internal standard [39]. For this work, we validated the assay as indicative of nitrogenase activity using several methods. Cultures that were not exposed to acetylene gas (undosed controls) had no detectable ethylene (Fig. 3). There was a concurrent drop in acetylene concentration with increasing concentrations of ethylene, further indicating that the ethylene was from the reduction of acetylene (data not shown). Furthermore, the WP5 nitrogenase mutant had no activity, further supporting the validity of the assay as a proxy for nitrogenase activity.

Regarding the negative ARA results from most of the Hawaiian plant samples, in this study, we sampled only a small section of shoots of Hawaiian plants, made extracts, and plated only a small volume for the screening test. Some of the other plant extracts did exhibit very low activity, and perhaps this low level would be sufficient for a slow-growing plant. Furthermore, because of the small amount of shoot tissue sampled, we cannot rule out the presence of nitrogen-fixing endophytes in any of the plants sampled. The distribution of endophytes is known not to be uniform. Alternatively, plants could also obtain their nitrogen from root endophytic diazotrophs, from rhizospheric diazotrophs, or simply from other members of the microbiota in the extract that did not end up in the assay vial. A metagenomic analysis of the whole plants would help identify the locations of any associated nitrogen fixers. For this study, we chose to focus on the shoot samples with very high activity for our further analysis.

The majority of research on microbial interactions has focused on negative interactions such as competition and antimicrobial production, and only recently has the potential for positive microbe–microbe interactions been investigated [7, 8, 10, 40]. As we strive toward more sustainable agriculture, we can learn much from natural systems where plants can thrive despite abiotic and biotic challenges. In environments such as the primary substrates resulting from volcanic eruptions, glacial retreats, and flooding, colonizing plants depend on effective partnerships with microorganisms for nutrient acquisition. These pioneering plants host extraordinarily diverse ecosystems within, the interplay of which we are only just beginning to explore.

## Supplementary Material

Revised_10Dec2024_Supplementary_Figures_File_ycae158

Supplemental_Table_1_REV_ycae158

Supplemental_Tables_2-10_ycae158

Revised_10Dec2024_Non_Data_Supplementary_Data_Files_ycae158

## Data Availability

The draft genome assemblies for the strains used in this study were deposited in the NCBI database under the BioProjects PRJNA1058978 and PRJNA1059640. The datasets, R scripts, TYGS reports, and the statistical analyses are available in the figshare repository (https://www.figshare.com), DOI: https://doi.org/10.6084/m9.figshare.c.7002321.
