# Supplementary figures and images for: Bacterial synergies amplify nitrogenase activity in diverse systems
Source: ISME Commun. 2024 Dec 12;4(1):ycae158. doi: 10.1093/ismeco/ycae158 (PMC11684072; doi:10.1093/ismeco/ycae158)

**Supplementary Figures**

**Figure S1**

**Figure S2**


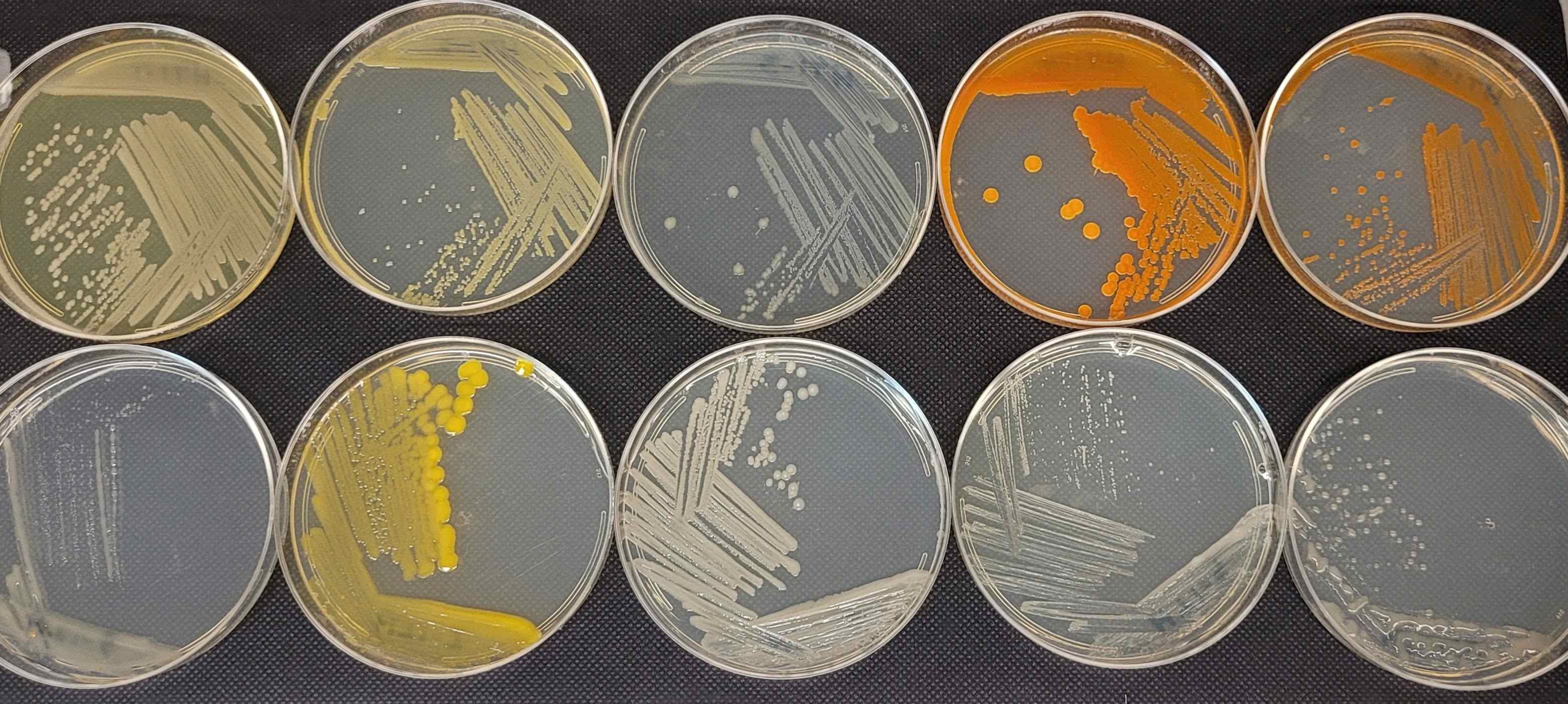


**Figure S3**

**Figure S4**

**Figure S5**


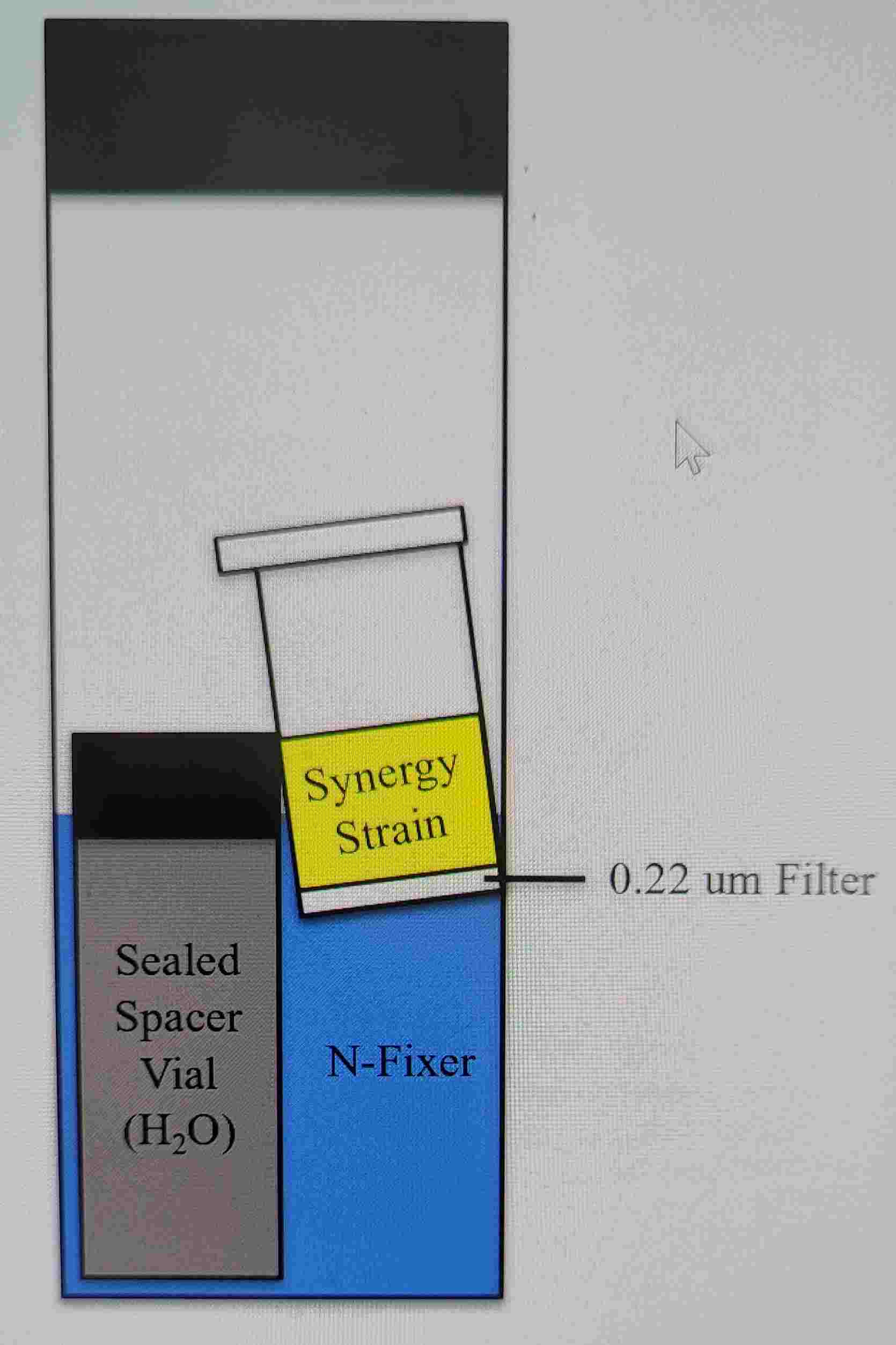

Supplement: Revised_10Dec2024_Supplementary_Figures_File_ycae158 [file revised_10dec2024_supplementary_figures_file_ycae158.docx]
