# Supplementary material for: Bacterial synergies amplify nitrogenase activity in diverse systems
Source: ISME Commun. 2024 Dec 12;4(1):ycae158. doi: 10.1093/ismeco/ycae158 (PMC11684072; doi:10.1093/ismeco/ycae158)
Supplement: Supplemental_Table_1_REV_ycae158 [file supplemental_table_1_rev_ycae158.pdf]

Table S1a. Metadata for the strains used in this study, including taxonomic classification, NCBI accessions, assembly and annotation, and isolate provenance.

BV-BRC[1]	Taxonomic Classification[2]					NCBI Accessions[3]	
**Genome ID**	**Taxon ID**	**Class**	**Genus**	**Species**	**Strain**	**Bioproject**	**Biosample**
6.53	6	Alphaproteobacteria	*Azorhizobium*	sp.	HT1-9	PRJNA1058978	SAMN39201417
	6	Alphaproteobacteria	*Azorhizobium*	sp.	HT1-6		
191.91	191	Alphaproteobacteria	*Azospirillum*	sp.	11R-A	PRJNA1058978	SAMN39201418
41275.76	41275	Alphaproteobacteria	*Brevundimonas*	sp.	HT1-5	PRJNA1058978	SAMN39201419
447237.14	447237	Actinomycetes	*Frondihabitans*	sp.	4ASC-45	PRJNA1058978	SAMN39201420
881616.28	881616	Actinomycetes	*Herbiconiux*	sp.	11R-BC	PRJNA1058978	SAMN39201421
34037.159	34037	Gammaproteobacteria	*Rahnella*	*aceris*	WP5	PRJNA247595	SAMN02787083
379.88	379	Alphaproteobacteria	*Rhizobium*	*wenxiniae*	HT1-8	PRJNA1058978	SAMN39201425
379.881	379	Alphaproteobacteria	*Rhizobium*	sp.	HT1-10	PRJNA1058978	SAMN39201426
165695.239	165695	Alphaproteobacteria	*Sphingobium*	sp.	11R-BB	PRJNA1058978	SAMN39201427
165695.238	165695	Alphaproteobacteria	*Sphingobium*	sp.	HT1-2	PRJNA1058978	SAMN39201428
165695.241	165695	Alphaproteobacteria	*Sphingobium*	sp.	WW5	PRJNA1059640	SAMN39212602
13687.293	13687	Alphaproteobacteria	*Sphingomonas*	sp.	4RDLI-65	PRJNA1058978	SAMN39201429

1 Bacterial and Viral Bioinformatics Resources Center (BV-BRC v3.34.11) (Barrett et al. 2012)

2 Type (Strain) Genome Server (TYGS v391) (Meier-Kolthoff & M. Göker 2019)

3 National Center for Biotechnology Information (NCBI) (Barrett et al. 2012)

Table S1a (continued) Metadata for the strains used in this study, including taxonomic classification, NCBI accessions, assembly and annotati

Strain				Assembly[4,5] and Annotation[6]						CheckM[7]	
**Genus**	**Strain**	**Center**	**Platform**	**Size (Mb)**	**Cov.**	**Contigs >=1000**	**N50 kbp**	**GC%**	**CDS**	**Comp %**	**Cont %**
Azorhizobium	HT1-9	Azenta (Genewiz	Illumina MiSeq	6.47	60x	26	542,850	66.83	5903	99.1	1.8
Azorhizobium	HT1-6										
Azospirillum	11R-A	Novagene	Illumina NovaSeq 6000	7.87	150x	73	201,078	67.81	7174	100	0.5
Brevundimonas	HT1-5	Azenta (Genewiz	Illumina MiSeq	3.10	60x	7	511,494	66.25	3078	100	0
Frondihabitans	4ASC-45	Novagene	Illumina NovaSeq 6000	3.54	150x	16	316,373	67.52	3405	99.3	0
Herbiconiux	11R-BC	Novagene	Illumina NovaSeq 6000	4.09	150x	11	475,220	70.30	3755	99.7	0.3
Rahnella	WP5	DOE JGI	Illumina HiSeq 2000	5.44	60x	20	444,182	52.15	5230	100	0
Rhizobium	HT1-8	Azenta (Genewiz	Illumina MiSeq	6.45	60x	25	513,216	58.79	6491	99.8	0.3
Rhizobium	HT1-10	Azenta (Genewiz	Illumina MiSeq	5.33	60x	24	582,124	61.24	5297	98.4	1.6
Sphingobium	11R-BB	Novagene	Illumina NovaSeq 6000	5.54	150x	100	158,314	64.08	5532	100	0.3
Sphingobium	HT1-2	Azenta (Genewiz	Illumina MiSeq	5.39	60x	50	690,876	64.20	5354	100	0.4
Sphingobium	WW5	Intrinsyx	Illumina MiSeq + Oxford Nanopore MinION	5.72	80x	8	5,061,924	64.00	5635	100	0.3
Sphingomonas	4RDLI-65	Novagene	Illumina NovaSeq 6000	4.01	150x	13	540,359	66.06	3651	99.9	1

[4,5] All draft assemblies were completed using SPAdes v3.13.0 (Bankevitch et al. 2012) , except for strain WW5 which was completed with Unicycler v0.5.0 (Wicks et al. 2017)

[6] All strains were annotated using RASTtk (Brettlin et al. 2015)

[7] CheckM (Parks et al. 2015)

**Table S1b.** Provenance of isolates used in this study.					
**Genus**	**Strain**	**Host (tissue)**	**Tissue**	**Date Collected**	**Location**
Azorhizobium	HT1-9	unkown dicot	shoot	2019-05	Island of Hawai'i, HI, USA
Azorhizobium	HT1-6	unkown dicot	shoot	2019-05	Island of Hawai'i, HI, USA
Azospirillum	11R-A	*Populus trichocarpa*	root	2020-09	Western WA, USA
Brevundimonas	HT1-5	unkown dicot	shoot	2019-05	Island of Hawai'i, HI, USA
Frondihabitans	4ASC-45	*Populus tremuloides*	stem	2020-09	Eastern WA, USA
Herbiconiux	11R-BC	*Populus trichocarpa*	root	2020-09	Eastern WA, USA
Rahnella	WP5	*Populus trichocarpa*	stem	2002-09	Western WA, USA
Rhizobium	HT1-8	unkown dicot	shoot	2019-05	Island of Hawai'i, HI, USA
Rhizobium	HT1-10	unkown dicot	shoot	2019-05	Island of Hawai'i, HI, USA
Sphingobium	11R-BB	*Populus trichocarpa*	root	2020-09	Western WA, USA
Sphingobium	HT1-2	unkown dicot	shoot	2019-05	Island of Hawai'i, HI, USA
Sphingobium	WW5	*Salix sitchensis*	stem	2002-05	Western WA, USA
Sphingomonas	4RDLI-65	*Cornus sericea*	root	2020-09	Eastern WA, USA
