# Supplementary material for: Bacterial synergies amplify nitrogenase activity in diverse systems
Source: ISME Commun. 2024 Dec 12;4(1):ycae158. doi: 10.1093/ismeco/ycae158 (PMC11684072; doi:10.1093/ismeco/ycae158)
Supplement: Supplemental_Tables_2-10_ycae158 [file supplemental_tables_2-10_ycae158.pdf]

Table S2. Synergism in Diazotrophs Co-Isolates. Satistical analysis of acetylene reduction assays ARA for the diazotroph *Azorhizobium* sp. HT1-9. Mean parts-per-million ppm, n = 3

Diazotroph	Group	Mean ppm	±SD	Min	Max
No diazotroph	HT1-2, HT1-5	0.00	0.00	0.00	0.00
No diazotroph	HT1-8, HT1-10	0.00	0.00	0.00	0.00
No diazotroph	HT1-2, HT1-5, HT1-8, HT1-10	0.00	0.00	0.00	0.00
Azorhizobium sp. HT1-9	HT1-6	41.02	13.47	26.80	53.59
Azorhizobium sp. HT1-9	HT1-2, HT1-6	269.42	5.05	263.63	272.89
Azorhizobium sp. HT1-9	HT1-5, HT1-6	115.53	8.39	107.14	123.91
Azorhizobium sp. HT1-9	HT1-2, HT1-5, HT1-6	241.70	9.21	236.05	252.33

one-way ANOVA, HT1-9

term	df	sumsq	meansq	statistic	p.value
treatment	3	103773.83	34591.3	382.13	5.66E-09
Residuals	8	724.18	90.52		

Pairwise Comparisons Bonferroni

contrast	estimate	SE	df	t.ratio	p.value
(HT1-9, HT1-6) + (HT1-2) vs. (HT1-9, HT1-6)	-228.4	7.77	8	-29.4	1.16E-08
(HT1-9, HT1-6) + (HT1-5) vs. (HT1-9, HT1-6)	-74.51	7.77	8	-9.59	6.95E-05
(HT1-9, HT1-6) + (HT1-2, HT1-5) vs. (HT1-9, HT1-6)	-200.68	7.77	8	-25.83	3.25E-08
(HT1-9, HT1-6) + (HT1-2) vs. (HT1-9, HT1-6) + HT1-5	153.90	7.77	8	19.81	2.63E-07
(HT1-9, HT1-6) + (HT1-2) vs. (HT1-9, HT1-6) + (HT1-2, HT1-5)	27.72	7.77	8	3.57	0.044
(HT1-9, HT1-6) +(HT1-5) vs. (HT1-9, HT1-6) + (HT1-2, HT1-5)	-126.18	7.77	8	-16.24	1.25E-06

Pairwise Effect Size Cohen's d

contrast	effect.size	SE	df	lower.CL	upper.CL
(HT1-9, HT1-6) + (HT1-2) vs. (HT1-9, HT1-6)	-24.01	6.06	8	-37.97	-10.04
(HT1-9, HT1-6) + (HT1-5) vs. (HT1-9, HT1-6)	-7.83	2.12	8	-12.72	-2.94
(HT1-9, HT1-6) + (HT1-2, HT1-5) vs. (HT1-9, HT1-6)	-21.09	5.34	8	-33.4	-8.79
(HT1-9, HT1-6) + (HT1-2) vs. (HT1-9, HT1-6) + HT1-5	16.18	4.13	8	6.66	25.69
(HT1-9, HT1-6) + (HT1-2) vs. (HT1-9, HT1-6) + (HT1-2, HT1-5)	2.91	1.09	8	0.39	5.44
(HT1-9, HT1-6) +(HT1-5) vs. (HT1-9, HT1-6) + (HT1-2, HT1-5)	-13.26	3.41	8	-21.14	-5.39

Table S3. Confirmation of Synergism. Statistical analysis of acetylene reduction assays (ARA) for the diazotrophs *Azorhizobium sp.* HT1-9, *Rahnella aceris* sp. WP5, *Azospirillum* sp. 11R-A. Mean parts-per-million (ppm), n = 3

Diazotroph	Group	Mean ppm	±SD	Min	Max
No diazotroph	*Sphingobium* sp. HT1-2	0.00	0.00	0.00	0.00
No diazotroph	*Sphingobium* sp. WW5	0.00	0.00	0.00	0.00
No diazotroph	*Sphingibium* sp. 11R-BB	0.00	0.00	0.00	0.00
Azorhizobium sp. HT1-9	Nitrogen free media, undosed	0.00	0.00	0.00	0.00
Azorhizobium sp. HT1-9	Nitrogen free media	170.90	11.78	159.17	182.72
Azorhizobium sp. HT1-9	*Sphingobium* sp. HT1-2	385.05	9.09	377.84	395.26
Rahnella aceris sp. WP5	Nitrogen free media, undosed	0.00	0.00	0.00	0.00
Rahnella aceris sp. WP5	Nitrogen free media	5.20	0.14	5.05	5.32
Rahnella aceris sp. WP5	*Sphingobium* sp. WW5	25.60	0.63	24.87	26.01
Azospirillum sp. 11R-A	Nitrogen free media, undosed	0.00	0.00	0.00	0.00
Azospirillum sp. 11R-A	Nitrogen free media	532.85	37.28	491.48	563.81
Azospirillum sp. 11R-A	*Sphingibium* sp. 11R-BB	728.60	45.77	689.74	779.05

one-way ANOVA, HT1-9

term	df	sumsq	meansq	statistic	p.value
treatment	1	68789.35	68789.35	621.73	1.54E-05
Residuals	4	442.57	110.64		

one-way ANOVA, WP5

term	df	sumsq	meansq	statistic	p.value
treatment	1	623.90	623.90	3009.93	6.61E-07
Residuals	4	0.83	0.21		

one-way ANOVA, 11R-A

term	df	sumsq	meansq	statistic	p.value
treatment	1	57475.20	57475.20	32.99	0.005
Residuals	4	6968.43	1742.11		

Table S3 (cont.). Confirmation of Synergism. Statistical analyses of acetylene reduction assays (ARA) for the diazotrophs *Azorhizobium sp.* HT1-9, *Rahnella aceris* sp. WP5, *Azospirillum* sp. 11R-A. Mean parts-per-million (ppm), n = 3

Pairwise Comparisons (Bonferroni), HT1-9					
**contrast**	**estimate**	**SE**	**df**	**t.ratio**	**p.value**
NFM - (HT1-2)	-214.15	8.59	4	-24.93	1.54E-05

Pairwise Comparisons (Bonferroni), WP5					
**contrast**	**estimate**	**SE**	**df**	**t.ratio**	**p.value**
NFM - WW5	-20.39	0.37	4	-54.86	6.61E-07

Pairwise Comparisons (Bonferroni), 11R-A					
**contrast**	**estimate**	**SE**	**df**	**t.ratio**	**p.value**
NFM - (11R-BB)	-195.75	34.08	4	-5.74	0.005

Pairwise Effect Size (Cohen's d), HT1-9					
**contrast**	**effect.size**	**SE**	**df**	**lower.CL**	**upper.CL**
(NFM - (HT1-2))	-20.36	7.24	4	-40.47	-0.25

Pairwise Effect Size (Cohen's d), WP5					
**contrast**	**effect.size**	**SE**	**df**	**lower.CL**	**upper.CL**
(NFM - WW5)	-44.8	15.86	4	-88.83	-0.76

Pairwise Effect Size (Cohen's d), 11R-A					
**contrast**	**effect.size**	**SE**	**df**	**lower.CL**	**upper.CL**
(NFM - (11R-BB))	-4.69	1.85	4	-9.82	0.44

Table S4. Heat-Killed Synergists and Specificity of the Synergism. Statistics analysis of acetylene reduction assays (ARA) for the diazotroph *Rahnella aceris* sp. WP5. Mean parts-per-million (ppm), n = 5

Diazotroph	Group	Mean ppm	±SD	Min	Max
Rahnella aceris sp. WP5	Nitrogen free media	0.41	0.27	0.13	0.70
Rahnella aceris sp. WP5	*E. coli* DH5a	0.43	0.23	0.24	0.81
Rahnella aceris sp. WP5	*S. cerevisiae*	0.12	0.05	0.06	0.20
Rahnella aceris sp. WP5	*Frondihabitans* sp. 4ASC-45	2.78	1.35	1.33	4.82
Rahnella aceris sp. WP5	*Sphingomonas* sp. 4RDLI-65	0.58	0.06	0.48	0.64
Rahnella aceris sp. WP5	*Rahnella aceris* WP5, nifH mutant	0.11	0.04	0.05	0.15
Rahnella aceris sp. WP5	*Sphingobium* sp. HT1-2	28.61	9.40	16.44	39.39
Rahnella aceris sp. WP5	*Sphingibium* sp. 11R-BB	20.02	8.30	8.12	28.52
Rahnella aceris sp. WP5	*Sphingobium* sp. WW5	20.97	3.79	16.02	24.79
Rahnella aceris sp. WP5	Heat-killed *Sphingobium* sp. HT1-2	0.53	0.33	0.20	1.04
Rahnella aceris sp. WP5	Heat-killed *Sphingobium* sp. 11R-BB	0.51	0.22	0.21	0.74
Rahnella aceris sp. WP5	Heat-killed *Sphingobium* sp. WW5	0.33	0.18	0.15	0.58

one-way ANOVA, WP5

term	df	sumsq	meansq	statistic	p.value
treatment	11	5970.66	542.79	37.51	8.44E-20
Residuals	48	694.64	14.47		

Pairwise Comparisons (Bonferroni)

contrast	estimate	SE	df	t.ratio	p.value
NFM - DH5a	-0.02	2.41	48	-0.01	1.00
NFM - S.cer	0.29	2.41	48	0.12	1.00
NFM - (4ASC-45)	-2.38	2.41	48	-0.99	1.00
NFM - (4RDLI-65)	-0.18	2.41	48	-0.07	1.00
NFM - WP5mut	0.30	2.41	48	0.12	1.00
NFM - (HT1-2)	-28.20	2.41	48	-11.72	0.00
NFM - (11R-BB)	-19.62	2.41	48	-8.15	8.32E-09
NFM - WW5	-20.56	2.41	48	-8.55	2.15E-09
NFM - (HK-HT1-2)	-0.12	2.41	48	-0.05	1.00
NFM - (HK-11R-BB)	-0.10	2.41	48	-0.04	1.00
NFM - (HK-WW5)	0.07	2.41	48	0.03	1.00
DH5a - S.cer	0.31	2.41	48	0.13	1.00
DH5a - (4ASC-45)	-2.36	2.41	48	-0.98	1.00
DH5a - (4RDLI-65)	-0.16	2.41	48	-0.07	1.00
DH5a - WP5mut	0.32	2.41	48	0.13	1.00
DH5a - (HT1-2)	-28.18	2.41	48	-11.71	0.00
DH5a - (11R-BB)	-19.59	2.41	48	-8.14	8.57E-09
DH5a - WW5	-20.54	2.41	48	-8.54	2.22E-09
DH5a - (HK-HT1-2)	-0.10	2.41	48	-0.04	1.00
DH5a - (HK-11R-BB)	-0.08	2.41	48	-0.04	1.00
DH5a - (HK-WW5)	0.09	2.41	48	0.04	1.00
S.cer - (4ASC-45)	-2.66	2.41	48	-1.11	0.99
S.cer - (4RDLI-65)	-0.46	2.41	48	-0.19	1.00
S.cer - WP5mut	0.01	2.41	48	0.01	1.00

Table S4 (cont.). Heat-Killed Synergists and Specificity of the Synergism. Pairwise Bonferroni's correction post hoc tests. **A**cetylene reduction assays (ARA) for the diazotroph *Rahnella aceris* sp. WP5. Mean parts-per-million (ppm), n = 5

Pairwise Comparisons (Bonferroni)					
**contrast**	**estimate**	**SE**	**df**	**t.ratio**	**p.value**
S.cer - (HT1-2)	-28.49	2.41	48	-11.84	0.00
S.cer - (11R-BB)	-19.90	2.41	48	-8.27	5.53E-09
S.cer - WW5	-20.85	2.41	48	-8.66	1.43E-09
S.cer - (HK-HT1-2)	-0.41	2.41	48	-0.17	1.00
S.cer - (HK-11R-BB)	-0.39	2.41	48	-0.16	1.00
S.cer - (HK-WW5)	-0.21	2.41	48	-0.09	1.00
(4ASC-45) - (4RDLI-65)	2.20	2.41	48	0.91	1.00
(4ASC-45) - WP5mut	2.68	2.41	48	1.11	0.99
(4ASC-45) - (HT1-2)	-25.82	2.41	48	-10.73	0.00
(4ASC-45) - (11R-BB)	-17.24	2.41	48	-7.16	2.61E-07
(4ASC-45) - WW5	-18.18	2.41	48	-7.56	6.60E-08
(4ASC-45) - (HK-HT1-2)	2.25	2.41	48	0.94	1.00
(4ASC-45) - (HK-11R-BB)	2.27	2.41	48	0.94	1.00
(4ASC-45) - (HK-WW5)	2.45	2.41	48	1.02	1.00
(4RDLI-65) - WP5mut	0.47	2.41	48	0.20	1.00
(4RDLI-65) - (HT1-2)	-28.02	2.41	48	-11.65	0.00
(4RDLI-65) - (11R-BB)	-19.44	2.41	48	-8.08	1.07E-08
(4RDLI-65) - WW5	-20.38	2.41	48	-8.47	2.77E-09
(4RDLI-65) - (HK-HT1-2)	0.05	2.41	48	0.02	1.00
(4RDLI-65) - (HK-11R-BB)	0.07	2.41	48	0.03	1.00
(4RDLI-65) - (HK-WW5)	0.25	2.41	48	0.10	1.00
WP5mut - (HT1-2)	-28.50	2.41	48	-11.84	0.00
WP5mut - (11R-BB)	-19.91	2.41	48	-8.28	5.43E-09
WP5mut - WW5	-20.86	2.41	48	-8.67	1.41E-09
WP5mut - (HK-HT1-2)	-0.42	2.41	48	-0.18	1.00
WP5mut - (HK-11R-BB)	-0.40	2.41	48	-0.17	1.00
WP5mut - (HK-WW5)	-0.22	2.41	48	-0.09	1.00
(HT1-2) - (11R-BB)	8.59	2.41	48	3.57	0.04
(HT1-2) - WW5	7.64	2.41	48	3.18	0.09
(HT1-2) - (HK-HT1-2)	28.08	2.41	48	11.67	0.00
(HT1-2) - (HK-11R-BB)	28.10	2.41	48	11.68	0.00
(HT1-2) - (HK-WW5)	28.27	2.41	48	11.75	0.00
(11R-BB) - WW5	-0.95	2.41	48	-0.39	1.00
(11R-BB) - (HK-HT1-2)	19.49	2.41	48	8.10	9.95E-09
(11R-BB) - (HK-11R-BB)	19.51	2.41	48	8.11	9.67E-09
(11R-BB) - (HK-WW5)	19.69	2.41	48	8.18	7.50E-09
WW5 - (HK-HT1-2)	20.44	2.41	48	8.49	2.57E-09
WW5 - (HK-11R-BB)	20.46	2.41	48	8.50	2.50E-09
WW5 - (HK-WW5)	20.63	2.41	48	8.58	1.94E-09
(HK-HT1-2) - (HK-11R-BB)	0.02	2.41	48	0.01	1.00
(HK-HT1-2) - (HK-WW5)	0.20	2.41	48	0.08	1.00
(HK-11R-BB) - (HK-WW5)	0.18	2.41	48	0.07	1.00

Table S4 (cont.). Heat-Killed Synergists and Specificity of the Synergism. Pairwise Cohen's d effect size results. Acetylene reduction assays (ARA) for the diazotroph *Rahnella aceris* sp. WP5. Mean parts-per-million (ppm), n = 5

Pairwise Effect Size (Cohen's d)

contrast	effect.size	SE	df	lower.CL	upper.CL
(NFM - DH5a)	-0.01	0.63	48.00	-1.28	1.27
(NFM - S.cer)	0.07	0.63	48.00	-1.20	1.35
(NFM - (4ASC-45))	-0.63	0.64	48.00	-1.90	0.65
(NFM - (4RDLI-65))	-0.05	0.63	48.00	-1.32	1.22
(NFM - WP5mut)	0.08	0.63	48.00	-1.19	1.35
(NFM - (HT1-2))	-7.41	0.99	48.00	-9.40	-5.43
(NFM - (11R-BB))	-5.16	0.82	48.00	-6.81	-3.50
(NFM - WW5)	-5.40	0.84	48.00	-7.09	-3.72
(NFM - (HK-HT1-2))	-0.03	0.63	48.00	-1.30	1.24
(NFM - (HK-11R-BB))	-0.03	0.63	48.00	-1.30	1.24
(NFM - (HK-WW5))	0.02	0.63	48.00	-1.25	1.29
(DH5a - S.cer)	0.08	0.63	48.00	-1.19	1.35
(DH5a - (4ASC-45))	-0.62	0.64	48.00	-1.90	0.66
(DH5a - (4RDLI-65))	-0.04	0.63	48.00	-1.31	1.23
(DH5a - WP5mut)	0.08	0.63	48.00	-1.19	1.36
(DH5a - (HT1-2))	-7.41	0.99	48.00	-9.39	-5.43
(DH5a - (11R-BB))	-5.15	0.82	48.00	-6.80	-3.50
(DH5a - WW5)	-5.40	0.84	48.00	-7.09	-3.71
(DH5a - (HK-HT1-2))	-0.03	0.63	48.00	-1.30	1.24
(DH5a - (HK-11R-BB))	-0.02	0.63	48.00	-1.29	1.25
(DH5a - (HK-WW5))	0.02	0.63	48.00	-1.25	1.30
(S.cer - (4ASC-45))	-0.70	0.64	48.00	-1.98	0.58
(S.cer - (4RDLI-65))	-0.12	0.63	48.00	-1.39	1.15
(S.cer - WP5mut)	0.00	0.63	48.00	-1.27	1.27
(S.cer - (HT1-2))	-7.49	0.99	48.00	-9.48	-5.49
(S.cer - (11R-BB))	-5.23	0.83	48.00	-6.90	-3.57
(S.cer - WW5)	-5.48	0.84	48.00	-7.18	-3.78
(S.cer - (HK-HT1-2))	-0.11	0.63	48.00	-1.38	1.16
(S.cer - (HK-11R-BB))	-0.10	0.63	48.00	-1.37	1.17
(S.cer - (HK-WW5))	-0.06	0.63	48.00	-1.33	1.22
((4ASC-45) - (4RDLI-65))	0.58	0.64	48.00	-0.70	1.86
((4ASC-45) - WP5mut)	0.70	0.64	48.00	-0.58	1.98
((4ASC-45) - (HT1-2))	-6.79	0.94	48.00	-8.67	-4.90
((4ASC-45) - (11R-BB))	-4.53	0.78	48.00	-6.11	-2.96
((4ASC-45) - WW5)	-4.78	0.80	48.00	-6.39	-3.17
((4ASC-45) - (HK-HT1-2))	0.59	0.64	48.00	-0.68	1.87
((4ASC-45) - (HK-11R-BB))	0.60	0.64	48.00	-0.68	1.88
((4ASC-45) - (HK-WW5))	0.64	0.64	48.00	-0.63	1.92
((4RDLI-65) - WP5mut)	0.12	0.63	48.00	-1.15	1.40
((4RDLI-65) - (HT1-2))	-7.37	0.98	48.00	-9.34	-5.39
((4RDLI-65) - (11R-BB))	-5.11	0.82	48.00	-6.76	-3.46
((4RDLI-65) - WW5)	-5.36	0.84	48.00	-7.04	-3.68
((4RDLI-65) - (HK-HT1-2))	0.01	0.63	48.00	-1.26	1.29
((4RDLI-65) - (HK-11R-BB))	0.02	0.63	48.00	-1.25	1.29

Table S4 (cont.). Heat-Killed Synergists and Specificity of the Synergism. Pairwise Cohen's d effect size results. Acetylene reduction assays (ARA) for the diazotroph *Rahnella aceris* sp. WP5. Mean parts-per-million (ppm), n = 5

Pairwise Effect Size (Cohen's d)

contrast	effect.size	SE	df	lower.CL	upper.CL
((4RDLI-65) - (HK-WW5))	0.07	0.63	48.00	-1.21	1.34

(WP5mut - (HT1-2))	-7.49	0.99	48.00	-9.49	-5.50
(WP5mut - (11R-BB))	-5.23	0.83	48.00	-6.90	-3.57
(WP5mut - WW5)	-5.48	0.84	48.00	-7.18	-3.78
(WP5mut - (HK-HT1-2))	-0.11	0.63	48.00	-1.38	1.16
(WP5mut - (HK-11R-BB))	-0.11	0.63	48.00	-1.38	1.17
(WP5mut - (HK-WW5))	-0.06	0.63	48.00	-1.33	1.21
((HT1-2) - (11R-BB))	2.26	0.67	48.00	0.90	3.61
((HT1-2) - WW5)	2.01	0.66	48.00	0.67	3.35
((HT1-2) - (HK-HT1-2))	7.38	0.98	48.00	5.40	9.36
((HT1-2) - (HK-11R-BB))	7.39	0.98	48.00	5.41	9.36
((HT1-2) - (HK-WW5))	7.43	0.99	48.00	5.45	9.42
((11R-BB) - WW5)	-0.25	0.63	48.00	-1.52	1.02
((11R-BB) - (HK-HT1-2))	5.12	0.82	48.00	3.47	6.77
((11R-BB) - (HK-11R-BB))	5.13	0.82	48.00	3.48	6.78
((11R-BB) - (HK-WW5))	5.18	0.82	48.00	3.52	6.83
(WW5 - (HK-HT1-2))	5.37	0.84	48.00	3.69	7.05
(WW5 - (HK-11R-BB))	5.38	0.84	48.00	3.69	7.06
(WW5 - (HK-WW5))	5.42	0.84	48.00	3.73	7.11
((HK-HT1-2) - (HK-11R-BB))	0.01	0.63	48.00	-1.27	1.28
((HK-HT1-2) - (HK-WW5))	0.05	0.63	48.00	-1.22	1.32
((HK-11R-BB) - (HK-WW5))	0.05	0.63	48.00	-1.23	1.32

Table S5. Heat-Killed Synergists. Descriptive statistics from acetylene reduction assays (ARA) for the diazotrophs *Azorhizobium sp.* HT1-9, *Rahnella aceris* sp. WP5, *Azospirillum* sp. 11R-A. Mean parts-per-million (ppm), n = 5

Diazotroph	Group	Mean ppm	±SD	Min	Max
Rahnella aceris sp. WP5	Nitrogen free media	0.41	0.27	0.13	0.70
Rahnella aceris sp. WP5	Heat-killed *Sphingobium* sp. WW5	0.33	0.18	0.15	0.58
Rahnella aceris sp. WP5	Heat-killed *Sphingobium* sp. HT1-2	0.53	0.33	0.20	1.04
Rahnella aceris sp. WP5	Heat-killed *Sphingobium* sp. 11R-BB	0.51	0.22	0.21	0.74
Rahnella aceris sp. WP5	*Sphingobium* sp. HT1-2	28.61	9.40	16.44	39.39
Rahnella aceris sp. WP5	*Sphingobium* sp. WW5	20.97	3.79	16.02	24.79
Rahnella aceris sp. WP5	*Sphingibium* sp. 11R-BB	20.02	8.30	8.12	28.52
Azorhizobium sp. HT1-9	Nitrogen free media	101.86	8.13	92.34	111.09
Azorhizobium sp. HT1-9	Heat-killed *Sphingobium* sp. WW5	101.56	7.74	93.19	114.28
Azorhizobium sp. HT1-9	Heat-killed *Sphingobium* sp. HT1-2	92.95	12.33	78.70	108.17
Azorhizobium sp. HT1-9	Heat-killed *Sphingobium* sp. 11R-BB	93.82	16.70	67.71	108.40
Azorhizobium sp. HT1-9	*Sphingobium* sp. HT1-2	287.55	8.64	276.95	297.67
Azorhizobium sp. HT1-9	*Sphingobium* sp. WW5	184.83	17.07	163.62	202.87
Azorhizobium sp. HT1-9	*Sphingibium* sp. 11R-BB	182.00	9.21	172.95	193.89
Azospirillum sp. 11R-A	Nitrogen free media	466.34	28.70	425.11	504.76
Azospirillum sp. 11R-A	Heat-killed *Sphingobium* sp. WW5	497.92	27.63	468.25	541.17
Azospirillum sp. 11R-A	Heat-killed *Sphingobium* sp. HT1-2	532.41	66.93	470.33	640.68
Azospirillum sp. 11R-A	Heat-killed *Sphingobium* sp. 11R-BB	536.14	40.56	504.32	583.30
Azospirillum sp. 11R-A	*Sphingobium* sp. HT1-2	815.75	19.59	798.55	844.31
Azospirillum sp. 11R-A	*Sphingobium* sp. WW5	684.00	20.10	654.28	710.28
Azospirillum sp. 11R-A	*Sphingibium* sp. 11R-BB	679.16	17.00	656.64	699.47

one-way ANOVA, HT1-9

term	df	sumsq	meansq	statistic	p.value
treatment	6	161136.13	26856.02	186.5	3.06E-21
Residuals	28	4031.90	144.00		

one-way ANOVA, 11R-A

term	df	sumsq	meansq	statistic	p.value
treatment	6	483899.35	80649.89	64.25	4.49E-15
Residuals	28	35148.15	1255.29		

Table S5 (cont.). Heat-Killed Synergists. Pairwise Bonferroni's correction post hoc tests, **A**cetylene reduction assays (ARA) for the diazotrophs *Azorhizobium sp.* HT1-9, *Rahnella aceris* sp. WP5, *Azospirillum* sp. 11R-A. Mean parts-per-million (ppm), n = 5

Pairwise Comparisons (Bonferroni), HT1-9					
**contrast**	**estimate**	**SE**	**df**	**t.ratio**	**p.value**
NFM - (HK-WW5)	0.30	7.59	28	0.04	1.000
NFM - (HK-HT1-2)	8.91	7.59	28	1.17	1.000
NFM - (HK-11R-BB)	8.03	7.59	28	1.06	1.000
NFM - (HT1-2)	-185.69	7.59	28	-24.47	4.05E-19
NFM - WW5	-82.97	7.59	28	-10.93	2.72E-10
NFM - (11R-BB)	-80.14	7.59	28	-10.56	6.00E-10
(HK-WW5) - (HK-HT1-2)	8.62	7.59	28	1.14	1.000
(HK-WW5) - (HK-11R-BB)	7.74	7.59	28	1.02	1.000
(HK-WW5) - (HT1-2)	-185.99	7.59	28	-24.51	3.89E-19
(HK-WW5) - WW5	-83.27	7.59	28	-10.97	2.51E-10
(HK-WW5) - (11R-BB)	-80.44	7.59	28	-10.60	5.52E-10
(HK-HT1-2) - (HK-11R-BB)	-0.88	7.59	28	-0.12	1.000
(HK-HT1-2) - (HT1-2)	-194.60	7.59	28	-25.64	1.15E-19
(HK-HT1-2) - WW5	-91.88	7.59	28	-12.11	2.54E-11
(HK-HT1-2) - (11R-BB)	-89.05	7.59	28	-11.73	5.30E-11
(HK-11R-BB) - (HT1-2)	-193.73	7.59	28	-25.53	1.30E-19
(HK-11R-BB) - WW5	-91.01	7.59	28	-11.99	3.18E-11
(HK-11R-BB) - (11R-BB)	-88.17	7.59	28	-11.62	6.68E-11
(HT1-2) - WW5	102.72	7.59	28	13.53	1.74E-12
(HT1-2) - (11R-BB)	105.55	7.59	28	13.91	8.93E-13
WW5 - (11R-BB)	2.83	7.59	28	0.37	1.000

Pairwise Comparisons (Bonferroni), 11R-A					
**contrast**	**estimate**	**SE**	**df**	**t.ratio**	**p.value**
NFM - (HK-WW5)	-31.57	22.41	28	-1.41	1.000
NFM - (HK-HT1-2)	-66.07	22.41	28	-2.95	0.134
NFM - (HK-11R-BB)	-69.80	22.41	28	-3.11	0.089
NFM - (HT1-2)	-349.40	22.41	28	-15.59	5.18E-14
NFM - WW5	-217.66	22.41	28	-9.71	3.83E-09
NFM - (11R-BB)	-212.81	22.41	28	-9.50	6.23E-09
(HK-WW5) - (HK-HT1-2)	-34.49	22.41	28	-1.54	1.000
(HK-WW5) - (HK-11R-BB)	-38.22	22.41	28	-1.71	1.000
(HK-WW5) - (HT1-2)	-317.83	22.41	28	-14.18	5.51E-13
(HK-WW5) - WW5	-186.09	22.41	28	-8.30	1.03E-07
(HK-WW5) - (11R-BB)	-181.24	22.41	28	-8.09	1.75E-07
(HK-HT1-2) - (HK-11R-BB)	-3.73	22.41	28	-0.17	1.000
(HK-HT1-2) - (HT1-2)	-283.33	22.41	28	-12.64	9.02E-12
(HK-HT1-2) - WW5	-151.59	22.41	28	-6.77	5.04E-06
(HK-HT1-2) - (11R-BB)	-146.75	22.41	28	-6.55	8.89E-06
(HK-11R-BB) - (HT1-2)	-279.60	22.41	28	-12.48	1.24E-11
(HK-11R-BB) - WW5	-147.86	22.41	28	-6.60	7.80E-06
(HK-11R-BB) - (11R-BB)	-143.02	22.41	28	-6.38	1.38E-05
(HT1-2) - WW5	131.74	22.41	28	5.88	5.32E-05
(HT1-2) - (11R-BB)	136.59	22.41	28	6.10	2.97E-05
WW5 - (11R-BB)	4.84	22.41	28	0.22	1.000

Table S5 (cont.). Heat-Killed Synergists. Pairwise Cohen's d effect size results. **A**cetylene reduction assays (ARA) for the diazotrophs *Azorhizobium sp.* HT1-9, *Rahnella aceris* sp. WP5, *Azospirillum* sp. 11R-A. Mean parts-per-million (ppm), n = 5

contrast	effect.size	SE	df	lower.CL	upper.CL
(NFM - (HK-WW5))	0.02	0.63	28	-1.27	1.32
(NFM - (HK-HT1-2))	0.74	0.64	28	-0.57	2.05
(NFM - (HK-11R-BB))	0.67	0.64	28	-0.64	1.98
(NFM - (HT1-2))	-15.47	2.16	28	-19.9	-11.04
(NFM - WW5)	-6.91	1.12	28	-9.21	-4.62
(NFM - (11R-BB))	-6.68	1.09	28	-8.92	-4.44
((HK-WW5) - (HK-HT1-2))	0.72	0.64	28	-0.59	2.03
((HK-WW5) - (HK-11R-BB))	0.64	0.64	28	-0.66	1.95
((HK-WW5) - (HT1-2))	-15.50	2.17	28	-19.94	-11.06
((HK-WW5) - WW5)	-6.94	1.12	28	-9.24	-4.64
((HK-WW5) - (11R-BB))	-6.70	1.10	28	-8.95	-4.46
((HK-HT1-2) - (HK-11R-BB))	-0.07	0.63	28	-1.37	1.22
((HK-HT1-2) - (HT1-2))	-16.22	2.26	28	-20.84	-11.59
((HK-HT1-2) - WW5)	-7.66	1.20	28	-10.12	-5.19
((HK-HT1-2) - (11R-BB))	-7.42	1.18	28	-9.83	-5.01
((HK-11R-BB) - (HT1-2))	-16.14	2.25	28	-20.75	-11.54
((HK-11R-BB) - WW5)	-7.58	1.19	28	-10.03	-5.14
((HK-11R-BB) - (11R-BB))	-7.35	1.17	28	-9.74	-4.96
((HT1-2) - WW5)	8.56	1.31	28	5.88	11.24
((HT1-2) - (11R-BB))	8.80	1.33	28	6.06	11.53
(WW5 - (11R-BB))	0.24	0.63	28	-1.06	1.53

Pairwise Effect Size (Cohen's d), 11R-A

contrast	effect.size	SE	df	lower.CL	upper.CL
(NFM - (HK-WW5))	-0.89	0.64	28	-2.21	0.43
(NFM - (HK-HT1-2))	-1.86	0.68	28	-3.26	-0.47
(NFM - (HK-11R-BB))	-1.97	0.69	28	-3.37	-0.57
(NFM - (HT1-2))	-9.86	1.46	28	-12.86	-6.87
(NFM - WW5)	-6.14	1.04	28	-8.27	-4.02
(NFM - (11R-BB))	-6.01	1.02	28	-8.10	-3.91
((HK-WW5) - (HK-HT1-2))	-0.97	0.65	28	-2.30	0.35
((HK-WW5) - (HK-11R-BB))	-1.08	0.65	28	-2.41	0.25
((HK-WW5) - (HT1-2))	-8.97	1.36	28	-11.75	-6.19
((HK-WW5) - WW5)	-5.25	0.94	28	-7.19	-3.32
((HK-WW5) - (11R-BB))	-5.12	0.93	28	-7.02	-3.21
((HK-HT1-2) - (HK-11R-BB))	-0.11	0.63	28	-1.40	1.19
((HK-HT1-2) - (HT1-2))	-8.00	1.24	28	-10.54	-5.45
((HK-HT1-2) - WW5)	-4.28	0.85	28	-6.03	-2.53
((HK-HT1-2) - (11R-BB))	-4.14	0.84	28	-5.86	-2.42
((HK-11R-BB) - (HT1-2))	-7.89	1.23	28	-10.41	-5.37
((HK-11R-BB) - WW5)	-4.17	0.84	28	-5.90	-2.45
((HK-11R-BB) - (11R-BB))	-4.04	0.83	28	-5.74	-2.33
((HT1-2) - WW5)	3.72	0.80	28	2.07	5.37
((HT1-2) - (11R-BB))	3.86	0.82	28	2.18	5.53
(WW5 - (11R-BB))	0.14	0.63	28	-1.16	1.43

Table S6 (cont.). Ratio Experiments. Descriptive statistics from acetylene reduction assays (ARA) for the diazotrophs *Azorhizobium sp.* HT1-9, *Rahnella aceris* sp. WP5, *Azospirillum* sp. 11R-A. Mean parts-per-million (ppm), n = 3

Diazotroph	Group	Ratios	Mean ppm	±SD	Min	Max
Azorhizobium sp. HT1-9	Nitrogen free media	NFM	239.31	8.86	230.41	248.14
Azorhizobium sp. HT1-9	*Sphingobium* sp. HT1-2	S01:D10	325.67	58.63	278.13	391.18
Azorhizobium sp. HT1-9	*Sphingobium* sp. HT1-2	S01:D05	325.75	48.75	271.11	364.80
Azorhizobium sp. HT1-9	*Sphingobium* sp. HT1-2	S01:D01	333.57	8.84	323.60	340.41
Azorhizobium sp. HT1-9	*Sphingobium* sp. HT1-2	S05:D01	386.01	28.95	366.90	419.31
Azorhizobium sp. HT1-9	*Sphingobium* sp. HT1-2	S10:D01	402.05	39.27	369.10	445.50
Rahnella aceris sp. WP5	Nitrogen free media	NFM	1.69	0.28	1.47	2.01
Rahnella aceris sp. WP5	*Sphingobium* sp. WW5	S01:D10	53.34	12.80	38.56	60.93
Rahnella aceris sp. WP5	*Sphingobium* sp. WW5	S01:D05	51.93	3.40	48.10	54.61
Rahnella aceris sp. WP5	*Sphingobium* sp. WW5	S01:D01	54.85	7.36	46.36	59.22
Rahnella aceris sp. WP5	*Sphingobium* sp. WW5	S05:D01	70.75	26.05	47.37	98.83
Rahnella aceris sp. WP5	*Sphingobium* sp. WW5	S10:D01	64.78	10.06	54.75	74.86
Azospirillum sp. 11R-A	Nitrogen free media	NFM	492.12	23.87	467.22	514.82
Azospirillum sp. 11R-A	*Herbiconiux* sp. 11R-BC	S01:D10	516.12	7.81	509.71	524.82
Azospirillum sp. 11R-A	*Herbiconiux* sp. 11R-BC	S01:D05	559.25	36.99	530.37	600.94
Azospirillum sp. 11R-A	*Herbiconiux* sp. 11R-BC	S01:D01	667.68	18.82	646.43	682.24
Azospirillum sp. 11R-A	*Herbiconiux* sp. 11R-BC	S05:D01	1124.60	146.16	1020.76	1291.74
Azospirillum sp. 11R-A	*Herbiconiux* sp. 11R-BC	S10:D01	1178.68	175.33	977.07	1295.48

one-way ANOVA, HT1-9

term	df	sumsq	meansq	statistic	p.value
ratios	5	49283.11	9856.62	7.08	0.003
Residuals	12	16702.71	1391.89		

one-way ANOVA, WP5

term	df	sumsq	meansq	statistic	p.value
ratios	5	9059.19	1811.84	10.77	4.16E-04
Residuals	12	2018.45	168.2		

one-way ANOVA, 11R-A

term	df	sumsq	meansq	statistic	p.value
ratios	5	1464621.33	292924.27	32.27	1.47E-06
Residuals	12	108911.78	9075.98		

Table S6 (cont.). Ratio Experiments. Pairwise Bonferroni's correction, post hoc tests. Acetylene reduction assays (ARA) for the diazotrophs *Azorhizobium sp.* HT1-9, *Rahnella aceris* sp. WP5, *Azospirillum* sp. 11R-A. Mean parts-per-million (ppm), n = 3

Pairwise Comparisons (Bonferroni), HT1-9					
**contrast**	**estimate**	**SE**	**df**	**t.ratio**	**p.value**
NFM - S01:D10	-86.36	30.46	12	-2.83	0.12
NFM - S01:D05	-86.44	30.46	12	-2.84	0.12
NFM - S01:D01	-94.26	30.46	12	-3.09	0.08
NFM - S05:D01	-146.70	30.46	12	-4.82	0.00
NFM - S10:D01	-162.74	30.46	12	-5.34	0.00
S01:D10 - S01:D05	-0.08	30.46	12	0.00	1.00
S01:D10 - S01:D01	-7.91	30.46	12	-0.26	1.00
S01:D10 - S05:D01	-60.34	30.46	12	-1.98	0.40
S01:D10 - S10:D01	-76.38	30.46	12	-2.51	0.20
S01:D05 - S01:D01	-7.82	30.46	12	-0.26	1.00
S01:D05 - S05:D01	-60.26	30.46	12	-1.98	0.41
S01:D05 - S10:D01	-76.30	30.46	12	-2.50	0.20
S01:D01 - S05:D01	-52.43	30.46	12	-1.72	0.54
S01:D01 - S10:D01	-68.48	30.46	12	-2.25	0.29
S05:D01 - S10:D01	-16.04	30.46	12	-0.53	0.99

Pairwise Comparisons (Bonferroni), WP5					
**contrast**	**estimate**	**SE**	**df**	**t.ratio**	**p.value**
NFM - S01:D10	-51.64	10.59	12	-4.88	0.006
NFM - S01:D05	-50.24	10.59	12	-4.74	0.007
NFM - S01:D01	-53.16	10.59	12	-5.02	0.004
NFM - S05:D01	-69.06	10.59	12	-6.52	4.268E-04
NFM - S10:D01	-63.08	10.59	12	-5.96	7.269E-04
S01:D10 - S01:D05	1.41	10.59	12	0.13	1.000
S01:D10 - S01:D01	-1.51	10.59	12	-0.14	1.000
S01:D10 - S05:D01	-17.41	10.59	12	-1.64	1.000
S01:D10 - S10:D01	-11.44	10.59	12	-1.08	1.000
S01:D05 - S01:D01	-2.92	10.59	12	-0.28	1.000
S01:D05 - S05:D01	-18.82	10.59	12	-1.78	1.000
S01:D05 - S10:D01	-12.85	10.59	12	-1.21	1.000
S01:D01 - S05:D01	-15.9	10.59	12	-1.5	1.000
S01:D01 - S10:D01	-9.92	10.59	12	-0.94	1.000
S05:D01 - S10:D01	5.98	10.59	12	0.56	1.000

Pairwise Comparisons (Bonferroni), 11R-A					
**contrast**	**estimate**	**SE**	**df**	**t.ratio**	**p.value**
NFM - S01:D10	-24.00	77.79	12	-0.31	1.000
NFM - S01:D05	-67.13	77.79	12	-0.86	0.950
NFM - S01:D01	-175.55	77.79	12	-2.26	0.652
NFM - S05:D01	-632.48	77.79	12	-8.13	4.77E-05
NFM - S10:D01	-686.56	77.79	12	-8.83	2.03E-05
S01:D10 - S01:D05	-43.13	77.79	12	-0.55	0.990
S01:D10 - S01:D01	-151.55	77.79	12	-1.95	1.000
S01:D10 - S05:D01	-608.48	77.79	12	-7.82	7.09E-05
S01:D10 - S10:D01	-662.56	77.79	12	-8.52	2.95E-05
S01:D05 - S01:D01	-108.42	77.79	12	-1.39	1.000
S01:D05 - S05:D01	-565.35	77.79	12	-7.27	1.49E-04
S01:D05 - S10:D01	-619.43	77.79	12	-7.96	5.91E-05
S01:D01 - S05:D01	-456.93	77.79	12	-5.87	0.001
S01:D01 - S10:D01	-511.00	77.79	12	-6.57	3.98E-04
S05:D01 - S10:D01	-54.08	77.79	12	-0.70	0.980

Table S6 (cont.). Ratio Experiments. Pairwise Cohen's d effect size results. **A**cetylene reduction assays (ARA) for the diazotrophs *Azorhizobium sp.* HT1-9, *Rahnella aceris* sp. WP5, *Azospirillum* sp. 11R-A. Mean parts-per-million (ppm), n = 3

Pairwise Effect Size (Cohen's d), HT1-9					
**contrast**	**effect.size**	**SE**	**df**	**lower.CL**	**upper.CL**
(NFM - S01:D10)	-2.31	0.94	12	-4.37	-0.26
(NFM - S01:D05)	-2.32	0.94	12	-4.37	-0.26
(NFM - S01:D01)	-2.53	0.97	12	-4.63	-0.42
(NFM - S05:D01)	-3.93	1.14	12	-6.43	-1.44
(NFM - S10:D01)	-4.36	1.21	12	-6.99	-1.73
(S01:D10 - S01:D05)	0.00	0.82	12	-1.78	1.78
(S01:D10 - S01:D01)	-0.21	0.82	12	-1.99	1.57
(S01:D10 - S05:D01)	-1.62	0.88	12	-3.54	0.30
(S01:D10 - S10:D01)	-2.05	0.92	12	-4.05	-0.05
(S01:D05 - S01:D01)	-0.21	0.82	12	-1.99	1.57
(S01:D05 - S05:D01)	-1.62	0.88	12	-3.53	0.30
(S01:D05 - S10:D01)	-2.05	0.92	12	-4.04	-0.05
(S01:D01 - S05:D01)	-1.41	0.87	12	-3.29	0.48
(S01:D01 - S10:D01)	-1.84	0.90	12	-3.79	0.12
(S05:D01 - S10:D01)	-0.43	0.82	12	-2.22	1.36

Pairwise Effect Size (Cohen's d), WP5					
**contrast**	**effect.size**	**SE**	**df**	**lower.CL**	**upper.CL**
(NFM - S01:D10)	-3.98	1.15	12	-6.49	-1.47
(NFM - S01:D05)	-3.87	1.14	12	-6.35	-1.40
(NFM - S01:D01)	-4.10	1.17	12	-6.65	-1.55
(NFM - S05:D01)	-5.32	1.36	12	-8.29	-2.36
(NFM - S10:D01)	-4.86	1.29	12	-7.66	-2.06
(S01:D10 - S01:D05)	0.11	0.82	12	-1.67	1.89
(S01:D10 - S01:D01)	-0.12	0.82	12	-1.90	1.66
(S01:D10 - S05:D01)	-1.34	0.86	12	-3.22	0.53
(S01:D10 - S10:D01)	-0.88	0.84	12	-2.70	0.94
(S01:D05 - S01:D01)	-0.23	0.82	12	-2.01	1.56
(S01:D05 - S05:D01)	-1.45	0.87	12	-3.34	0.44
(S01:D05 - S10:D01)	-0.99	0.84	12	-2.82	0.84
(S01:D01 - S05:D01)	-1.23	0.85	12	-3.09	0.63
(S01:D01 - S10:D01)	-0.77	0.83	12	-2.58	1.05
(S05:D01 - S10:D01)	0.46	0.82	12	-1.33	2.25

Pairwise Effect Size (Cohen's d), 11R-A					
**contrast**	**effect.size**	**SE**	**df**	**lower.CL**	**upper.CL**
(NFM - S01:D10)	-0.25	0.82	12	-2.03	1.53
(NFM - S01:D05)	-0.70	0.83	12	-2.51	1.10
(NFM - S01:D01)	-1.84	0.90	12	-3.80	0.12
(NFM - S05:D01)	-6.64	1.58	12	-10.09	-3.19
(NFM - S10:D01)	-7.21	1.68	12	-10.87	-3.54
(S01:D10 - S01:D05)	-0.45	0.82	12	-2.24	1.34
(S01:D10 - S01:D01)	-1.59	0.88	12	-3.51	0.32
(S01:D10 - S05:D01)	-6.39	1.54	12	-9.74	-3.04
(S01:D10 - S10:D01)	-6.95	1.64	12	-10.52	-3.39
(S01:D05 - S01:D01)	-1.14	0.85	12	-2.99	0.71
(S01:D05 - S05:D01)	-5.93	1.46	12	-9.12	-2.75
(S01:D05 - S10:D01)	-6.50	1.56	12	-9.90	-3.11
(S01:D01 - S05:D01)	-4.80	1.27	12	-7.57	-2.02
(S01:D01 - S10:D01)	-5.36	1.37	12	-8.34	-2.39
(S05:D01 - S10:D01)	-0.57	0.82	12	-2.36	1.23

Table S7. Physical separation of synergists-diazotrophs. Descriptive statistics from acetylene reduction assays (ARA) for the diazotrophs *Azorhizobium sp.* HT1-9, *Rahnella aceris* sp. WP5, *Azospirillum* sp. 11R-A. Data are the normalized mean parts-per-million (ppm), n = 3-9

Diazotroph	Group	Reps	Mean ppm	±SD	Min	Max
Azorhizobium sp. HT1-9	Nitrogen limited media	6	1.00	0.36	0.39	1.31
Azorhizobium sp. HT1-9	*Sphingobium* sp. WW5	3	5.34	0.08	5.27	5.43
Azorhizobium sp. HT1-9	*Sphingobium* sp. HT1-2	6	5.44	0.84	4.31	6.48
Azorhizobium sp. HT1-9	*Sphingibium* sp. 11R-BB	3	6.72	0.83	6.06	7.65
Rahnella aceris sp. WP5	Nitrogen limited media	6	1.00	0.59	0.00	1.60
Rahnella aceris sp. WP5	*Sphingobium* sp. WW5	3	0.93	0.33	0.64	1.29
Rahnella aceris sp. WP5	*Sphingobium* sp. HT1-2	3	0.84	0.42	0.53	1.32
Rahnella aceris sp. WP5	*Sphingibium* sp. 11R-BB	3	0.59	0.04	0.55	0.63
Azospirillum sp. 11R-A	Nitrogen limited media	9	1.00	0.58	0.01	1.62
Azospirillum sp. 11R-A	*Sphingobium* sp. WW5	3	0.74	0.09	0.65	0.82
Azospirillum sp. 11R-A	*Sphingobium* sp. HT1-2	3	3.11	1.44	1.51	4.30
Azospirillum sp. 11R-A	*Sphingibium* sp. 11R-BB	3	0.92	0.54	0.30	1.30

one-way ANOVA, HT1-9

term	df	sumsq	meansq	statistic	p.value
treatment	3	93.56	31.19	79.17	5.08E-09
Residuals	14	5.52	0.39		

one-way ANOVA, WP5

term	df	sumsq	meansq	statistic	p.value
treatment	3	0.36	0.12	0.56	0.654
Residuals	11	2.33	0.21		

one-way ANOVA, 11R-A

term	df	sumsq	meansq	statistic	p.value
treatment	3	11.97	3.99	7.55	0.003
Residuals	14	7.40	0.53		

Table S7 (cont.). Physical separation of synergists-diazotrophs. Pairwise Bonferroni's comparison, post hoc tests. **A**cetylene reduction assays (ARA) for the diazotrophs *Azorhizobium sp.* HT1-9, *Rahnella aceris* sp. WP5, *Azospirillum* sp. 11R-A. Data are the normalized mean parts-per-million (ppm), n = 3-9

contrast	estimate	SE	df	t.ratio	p.value
NL - WW5	-4.34	0.44	14	-9.77	7.46E-07
NL - (HT1-2)	-4.44	0.36	14	-12.24	4.34E-08
NL - (11R-BB)	-5.72	0.44	14	-12.90	2.22E-08
WW5 - (HT1-2)	-0.10	0.44	14	-0.23	1.000
WW5 - (11R-BB)	-1.39	0.51	14	-2.71	0.102
(HT1-2) - (11R-BB)	-1.29	0.44	14	-2.90	0.070

Pairwise Comparisons (Bonferroni), WP5

contrast	estimate	SE	df	t.ratio	p.value
NL - WW5	0.07	0.33	11	0.21	1.000
NL - (HT1-2)	0.16	0.33	11	0.48	1.000
NL - (11R-BB)	0.41	0.33	11	1.27	1.000
WW5 - (HT1-2)	0.09	0.38	11	0.23	1.000
WW5 - (11R-BB)	0.34	0.38	11	0.91	1.000
(HT1-2) - (11R-BB)	0.26	0.38	11	0.68	1.000

Pairwise Comparisons (Bonferroni), 11R-A

contrast	estimate	SE	df	t.ratio	p.value
NL - WW5	0.26	0.48	14	0.54	1.000
NL - (HT1-2)	-2.11	0.48	14	-4.35	0.004
NL - (11R-BB)	0.08	0.48	14	0.16	1.000
WW5 - (HT1-2)	-2.37	0.59	14	-3.99	0.008
WW5 - (11R-BB)	-0.18	0.59	14	-0.31	1.000
(HT1-2) - (11R-BB)	2.18	0.59	14	3.68	0.015

Table S7 (cont.). Physical separation of synergists-diazotrophs. Pairwise Cohen's d effect size results. **A**cetylene reduction assays (ARA) for the diazotrophs *Azorhizobium sp.* HT1-9, *Rahnella aceris* sp. WP5, *Azospirillum* sp. 11R-A. Data are the normalized mean parts-per-million (ppm), n = 3-9

Pairwise Effect Size (Cohen's d), HT1-9					
**contrast**	**effect.size**	**SE**	**df**	**lower.CL**	**upper.CL**
(NL - WW5)	-6.91	1.48	14	-10.09	-3.72
(NL - (HT1-2))	-7.07	1.46	14	-10.19	-3.95
(NL - (11R-BB))	-9.12	1.86	14	-13.11	-5.12
(WW5 - (HT1-2))	-0.16	0.71	14	-1.68	1.36
(WW5 - (11R-BB))	-2.21	0.92	14	-4.18	-0.24
((HT1-2) - (11R-BB))	-2.05	0.81	14	-3.78	-0.32

Pairwise Effect Size (Cohen's d), WP5					
**contrast**	**effect.size**	**SE**	**df**	**lower.CL**	**upper.CL**
(NL - WW5)	0.15	0.71	11	-1.41	1.71
(NL - (HT1-2))	0.34	0.71	11	-1.22	1.90
(NL - (11R-BB))	0.90	0.73	11	-0.71	2.51
(WW5 - (HT1-2))	0.19	0.82	11	-1.61	1.99
(WW5 - (11R-BB))	0.75	0.83	11	-1.09	2.58
((HT1-2) - (11R-BB))	0.56	0.83	11	-1.26	2.37

Pairwise Effect Size (Cohen's d), 11R-A					
**contrast**	**effect.size**	**SE**	**df**	**lower.CL**	**upper.CL**
(NL - WW5)	0.36	0.67	14	-1.08	1.79
(NL - (HT1-2))	-2.90	0.86	14	-4.75	-1.05
(NL - (11R-BB))	0.11	0.67	14	-1.33	1.54
(WW5 - (HT1-2))	-3.26	1.02	14	-5.45	-1.06
(WW5 - (11R-BB))	-0.25	0.82	14	-2.01	1.50
((HT1-2) - (11R-BB))	3.00	0.99	14	0.87	5.14

Table S8. Microoxic (1%) vs. Ambient Conditions. Descriptive statistics from acetylene reduction assays (ARA) for the diazotrophs *Azorhizobium sp.* HT1-9, *Rahnella aceris* sp. WP5, *Azospirillum* sp. 11R-A. Mean parts-per-million (ppm), n = 3.

Diazotroph	Group	Mean ppm	±SD	Min	Max
No diazotroph	*Sphingobium* sp. HT1-2	0.00	0.00	0.00	0.00
No diazotroph	*Sphingobium* sp. HT1-2, microoxic	0.00	0.00	0.00	0.00
No diazotroph	*Sphingobium* sp. WW5	0.00	0.00	0.00	0.00
No diazotroph	*Sphingobium* sp. WW5, microoxic	0.00	0.00	0.00	0.00
No diazotroph	*Herbiconiux* sp. 11R-BC	0.00	0.00	0.00	0.00
No diazotroph	*Herbiconiux* sp. 11R-BC, microoxic	0.00	0.00	0.00	0.00
Azorhizobium sp. HT1-9	Nitrogen free media	150.03	9.77	138.79	156.51
Azorhizobium sp. HT1-9	Nitrogen free media, microoxic	75.79	7.09	68.82	83.00
Azorhizobium sp. HT1-9	*Sphingobium* sp. HT1-2	293.90	4.78	288.52	297.66
Azorhizobium sp. HT1-9	*Sphingobium* sp. HT1-2, microoxic	86.39	3.70	83.96	90.64
Rahnella aceris sp. WP5	Nitrogen free media	3.57	1.66	1.66	4.59
Rahnella aceris sp. WP5	Nitrogen free media, microoxic	32.79	8.86	22.64	38.95
Rahnella aceris sp. WP5	*Sphingobium* sp. WW5	42.23	11.60	28.86	49.56
Rahnella aceris sp. WP5	*Sphingobium* sp. WW5, microoxic	47.07	5.29	43.12	53.08
Azospirillum sp. 11R-A	Nitrogen free media	456.24	9.97	444.73	462.41
Azospirillum sp. 11R-A	Nitrogen free media, microoxic	213.65	7.55	205.37	220.15
Azospirillum sp. 11R-A	*Herbiconiux* sp. 11R-BC	576.85	32.68	540.56	603.97
Azospirillum sp. 11R-A	*Herbiconiux* sp. 11R-BC, microoxic	224.92	20.84	202.02	242.76

one-way ANOVA, HT1-9

term	df	sumsq	meansq	statistic	p.value
treatment	3	90756.36	30252.1	663.52	6.31E-10
Residuals	8	364.75	45.59		

one-way ANOVA, WP5

term	df	sumsq	meansq	statistic	p.value
treatment	3	3417.24	1139.08	18.7	5.66E-04
Residuals	8	487.25	60.91		

one-way ANOVA, 11R-A

term	df	sumsq	meansq	statistic	p.value
treatment	3	287104.36	95701.5	230.78	4.17E-08
Residuals	8	3317.55	414.69		

Table S8 (cont.). Microoxic (1%) vs. Ambient Conditions. Pairwise Bonferroni's comparison post hoc tests. Acetylene reduction assays (ARA) for the diazotrophs *Azorhizobium sp.* HT1-9, *Rahnella aceris* sp. WP5, *Azospirillum* sp. 11R-A. Mean parts-per-million (ppm), n = 3.

Pairwise Comparisons (Bonferroni), HT1-9					
**contrast**	**estimate**	**SE**	**df**	**t.ratio**	**p.value**
NFM - NFM, microoxic	74.24	5.51	8	13.47	5.32E-06
NFM - (HT1-2)	-143.87	5.51	8	-26.10	3.00E-08
NFM - (HT1-2, microoxic)	63.64	5.51	8	11.54	1.73E-05
NFM, microoxic - (HT1-2)	-218.11	5.51	8	-39.56	1.10E-09
NFM, microoxic - (HT1-2, microoxic)	-10.60	5.51	8	-1.92	0.545
(HT1-2) - (HT1-2, microoxic)	207.51	5.51	8	37.64	1.64E-09

Pairwise Comparisons (Bonferroni), WP5					
**contrast**	**estimate**	**SE**	**df**	**t.ratio**	**p.value**
NFM - NFM, microoxic	-29.21	6.37	8	-4.58	0.011
NFM - WW5	-38.66	6.37	8	-6.07	0.002
NFM - WW5, microoxic	-43.5	6.37	8	-6.83	8.05E-04
NFM, microoxic - WW5	-9.45	6.37	8	-1.48	1.000
NFM, microoxic - WW5, microoxic	-14.28	6.37	8	-2.24	0.332
WW5 - WW5, microoxic	-4.84	6.37	8	-0.76	1.000

Pairwise Comparisons (Bonferroni), 11R-A					
**contrast**	**estimate**	**SE**	**df**	**t.ratio**	**p.value**
NFM - NFM, microoxic	242.58	16.63	8	14.59	2.87E-06
NFM - (11R-BC)	-120.62	16.63	8	-7.25	5.26E-04
NFM - (11R-BC, microoxic)	231.32	16.63	8	13.91	4.14E-06
NFM, microoxic - (11R-BC)	-363.2	16.63	8	-21.84	1.22E-08
NFM, microoxic - (11R-BC, microoxic)	-11.26	16.63	8	-0.68	1.000
(11R-BC) - (11R-BC, microoxic)	351.94	16.63	8	21.17	1.57E-07

Table S8 (cont.). Microoxic (1%) vs. Ambient Conditions. Pairwise Cohen's d effect size results. Acetylene reduction assays (ARA) for the diazotrophs *Azorhizobium sp.* HT1-9, *Rahnella aceris* sp. WP5, *Azospirillum* sp. 11R-A. Mean parts-per-million (ppm), n = 3.

Pairwise Effect Size (Cohen's d), HT1-9					
**contrast**	**effect.size**	**SE**	**df**	**lower.CL**	**upper.CL**
(NFM - NFM, microoxic)	11.00	2.87	8	4.38	17.61
(NFM - (HT1-2))	-21.31	5.39	8	-33.73	-8.88
(NFM - (HT1-2, microoxic))	9.43	2.49	8	3.67	15.18
(NFM, microoxic - (HT1-2))	-32.30	8.12	8	-51.02	-13.58
(NFM, microoxic - (HT1-2, microoxic))	-1.57	0.91	8	-3.66	0.52
((HT1-2) - (HT1-2, microoxic))	30.73	7.73	8	12.92	48.55

Pairwise Effect Size (Cohen's d), WP5					
**contrast**	**effect.size**	**SE**	**df**	**lower.CL**	**upper.CL**
(NFM - NFM, microoxic)	-3.74	1.24	8	-6.61	-0.88
(NFM - WW5)	-4.95	1.48	8	-8.37	-1.53
(NFM - WW5, microoxic)	-5.57	1.61	8	-9.30	-1.85
(NFM, microoxic - WW5)	-1.21	0.87	8	-3.22	0.80
(NFM, microoxic - WW5, microoxic)	-1.83	0.94	8	-3.99	0.33
(WW5 - WW5, microoxic)	-0.62	0.83	8	-2.54	1.30

Pairwise Effect Size (Cohen's d), 11R-A					
**contrast**	**effect.size**	**SE**	**df**	**lower.CL**	**upper.CL**
(NFM - NFM, microoxic)	11.91	3.09	8	4.79	19.03
(NFM - (11R-BC))	-5.92	1.69	8	-9.82	-2.02
(NFM - (11R-BC, microoxic))	11.36	2.95	8	4.55	18.17
(NFM, microoxic - (11R-BC))	-17.84	4.53	8	-28.29	-7.38
(NFM, microoxic - (11R-BC, microoxic))	-0.55	0.83	8	-2.46	1.36
((11R-BC) - (11R-BC, microoxic))	17.28	4.40	8	7.14	27.42

Table S9. Templates and Primers used in making pUC18-miniTn7T2-Gm-GFP

Fragment	Primer Name	Primer Sequence	Template	Final Construct
Vector	1-F	aacggtctgcgttgtcgggaagatgcgtgataccgaacaactccgcggcc	pUC18-miniTn7T2-Gm-mCherry	pUC18-miniTn7T2-Gm-GFP
	1-R	tattcaacgggaaacgtcttgctcgaggccgcgttacgccgtgggtcgat		
GFP	2-F	aacatcaaacatcgacccacggcgtaacgcggcctcgagcaagacgtttc	pBHR-GFP-Km	
	2-R	tcggcttcccggccgcggagttgttcggtatcacgcatcttcccgacaac		

Table S10. No increase in growth of diazotrophs with synergistic partners in nitrogen limited media. Statistical analysis of growth assays for the diazotrophs *Azorhizobium sp.* HT1-9, *Rahnella aceris* sp. WP5, *Azospirillum* sp. 11R-A in Nitrogen limited media with and without synergistic strains. Mean fluorescence (GFP, 489/520), n = 3

Diazotroph	Group	Hour	Mean GFP (489/520)	±SD	Min	Max
Azorhizobium sp. HT1-9	Nitrogen limited media	0	103.444	4.73	98.333	107.667
Azorhizobium sp. HT1-9	Nitrogen limited media	24	180.444	14.508	165.667	194.667
Azorhizobium sp. HT1-9	Nitrogen limited media	48	179.667	19.877	157.667	196.333
Azorhizobium sp. HT1-9	*Sphingibium* sp. 11R-BB	0	106.111	3.289	102.333	108.333
Azorhizobium sp. HT1-9	*Sphingibium* sp. 11R-BB	24	166.778	42.628	142	216
Azorhizobium sp. HT1-9	*Sphingibium* sp. 11R-BB	48	170.444	2.365	168.333	173
Azorhizobium sp. HT1-9	*Sphingobium* sp. HT1-2	0	109.111	7.074	101	114
Azorhizobium sp. HT1-9	*Sphingobium* sp. HT1-2	24	158.667	33.448	128	194.333
Azorhizobium sp. HT1-9	*Sphingobium* sp. HT1-2	48	204.778	57.203	165	270.333
Azorhizobium sp. HT1-9	*Sphingobium* sp. WW5	0	109.333	4.041	105.667	113.667
Azorhizobium sp. HT1-9	*Sphingobium* sp. WW5	24	199.778	90.458	141.667	304
Azorhizobium sp. HT1-9	*Sphingobium* sp. WW5	48	203.222	29.609	177	235.333
Rahnella aceris sp. WP5	Nitrogen limited media	0	470.556	153.042	322.333	628
Rahnella aceris sp. WP5	Nitrogen limited media	24	727.667	123.236	591	830.333
Rahnella aceris sp. WP5	Nitrogen limited media	48	701.667	33.716	672	738.333
Rahnella aceris sp. WP5	*Sphingibium* sp. 11R-BB	0	484.333	161.711	348	663
Rahnella aceris sp. WP5	*Sphingibium* sp. 11R-BB	24	724.556	75.39	655	804.667
Rahnella aceris sp. WP5	*Sphingibium* sp. 11R-BB	48	634.556	135.116	516	781.667
Rahnella aceris sp. WP5	*Sphingobium* sp. HT1-2	0	497.556	180.141	348.333	697.667
Rahnella aceris sp. WP5	*Sphingobium* sp. HT1-2	24	785.778	38.788	750	827
Rahnella aceris sp. WP5	*Sphingobium* sp. HT1-2	48	793.333	236.26	540	1007.667
Rahnella aceris sp. WP5	*Sphingobium* sp. WW5	0	467.111	159.367	350.333	648.667
Rahnella aceris sp. WP5	*Sphingobium* sp. WW5	24	724.556	87.449	642.667	816.667
Rahnella aceris sp. WP5	*Sphingobium* sp. WW5	48	701.333	108.307	579	785
Azospirillum sp. 11R-A	Nitrogen limited media	0	91.667	2.082	90	94
Azospirillum sp. 11R-A	Nitrogen limited media	24	252.778	99.412	138	311.667
Azospirillum sp. 11R-A	Nitrogen limited media	48	320	58.499	281.667	387.333
Azospirillum sp. 11R-A	*Sphingibium* sp. 11R-BB	0	93.444	0.77	93	94.333
Azospirillum sp. 11R-A	*Sphingibium* sp. 11R-BB	24	131.889	27.415	103.333	158
Azospirillum sp. 11R-A	*Sphingibium* sp. 11R-BB	48	174.333	51.265	121.333	223.667
Azospirillum sp. 11R-A	*Sphingobium* sp. HT1-2	0	95.444	5.621	89	99.333
Azospirillum sp. 11R-A	*Sphingobium* sp. HT1-2	24	176.222	72.491	113.667	255.667
Azospirillum sp. 11R-A	*Sphingobium* sp. HT1-2	48	285	139.682	165	438.333

Azospirillum sp. 11R-A	*Sphingobium* sp. WW5	0	96.444	3.025	93	98.667
Azospirillum sp. 11R-A	*Sphingobium* sp. WW5	24	165.667	73.902	106	248.333
Azospirillum sp. 11R-A	*Sphingobium* sp. WW5	48	224.333	97.167	137	329

Repeated Measures ANOVA (Synergistic partner vs Nitrogen limited media), HT1-9

hour	synergistic partner	estimate	SE	df	t.ratio	p.value
0	*Sphingibium* sp. 11R-BB	2.67	29.8	24	0.09	0.9969
0	*Sphingobium* sp. HT1-2	5.67	29.8	24	0.19	0.9857
0	*Sphingobium* sp. WW5	5.89	29.8	24	0.198	0.9846
24	*Sphingibium* sp. 11R-BB	-13.67	29.8	24	-0.459	0.9131
24	*Sphingobium* sp. HT1-2	-21.78	29.8	24	-0.731	0.7827
24	*Sphingobium* sp. WW5	19.33	29.8	24	0.649	0.8269
48	*Sphingibium* sp. 11R-BB	-9.22	29.8	24	-0.31	0.9611
48	*Sphingobium* sp. HT1-2	25.11	29.8	24	0.843	0.7178
48	*Sphingobium* sp. WW5	23.56	29.8	24	0.791	0.7487

Repeated Measures ANOVA (Synergistic partner vs Nitrogen limited media), WP5

hour	synergistic partner	estimate	SE	df	t.ratio	p.value
0	*Sphingibium* sp. 11R-BB	13.778	112	24	0.123	0.9941
0	*Sphingobium* sp. HT1-2	27	112	24	0.242	0.9766
0	*Sphingobium* sp. WW5	-3.444	112	24	-0.031	0.9996
24	*Sphingibium* sp. 11R-BB	-3.111	112	24	-0.028	0.9997
24	*Sphingobium* sp. HT1-2	58.111	112	24	0.52	0.8879
24	*Sphingobium* sp. WW5	-3.111	112	24	-0.028	0.9997
48	*Sphingibium* sp. 11R-BB	-67.111	112	24	-0.601	0.8509
48	*Sphingobium* sp. HT1-2	91.667	112	24	0.821	0.7308
48	*Sphingobium* sp. WW5	-0.333	112	24	-0.003	1

Repeated Measures ANOVA (Synergistic partner vs Nitrogen limited media), 11R-A

hour	synergistic partner	estimate	SE	df	t.ratio	p.value
0	*Sphingibium* sp. 11R-BB	1.78	56	24	0.032	0.9996
0	*Sphingobium* sp. HT1-2	3.78	56	24	0.067	0.9983
0	*Sphingobium* sp. WW5	4.78	56	24	0.085	0.9972
24	*Sphingibium* sp. 11R-BB	-120.89	56	24	-2.16	0.1054
24	*Sphingobium* sp. HT1-2	-76.56	56	24	-1.368	0.4011
24	*Sphingobium* sp. WW5	-87.11	56	24	-1.556	0.305
48	*Sphingibium* sp. 11R-BB	-145.67	56	24	-2.602	0.042
48	*Sphingobium* sp. HT1-2	-35	56	24	-0.625	0.8389

48	*Sphingobium* sp. WW5	-95.67	56	24	-1.709	0.2389
