# Supplementary material for: Bacterial synergies amplify nitrogenase activity in diverse systems
Source: ISME Commun. 2024 Dec 12;4(1):ycae158. doi: 10.1093/ismeco/ycae158 (PMC11684072; doi:10.1093/ismeco/ycae158)
Supplement: Revised_10Dec2024_Non_Data_Supplementary_Data_Files_ycae158 [file revised_10dec2024_non_data_supplementary_data_files_ycae158.pdf]

Bacterial synergies amplify nitrogenase activity in diverse systems

Running title: Synergies amplify nitrogenase activity

Andrew W. Sher, Robert J. Tournay, Emma Gomez-Rivas, and Sharon L. Doty

SUPPLEMENTARY DATA FILES

File Name	Description
Figure S1	Screen of Hawaiian plant samples by ARA
Figure S2	Photo of the HT1 strain diversity
Figure S3	ARA of HT1 Mix and HT1 diazotrophs
Figure S4	Example ARA screening of yellow-pigmented strains
Figure S5	Diagram of SpinX set up for separating strains
Table_S1_strain_metadata.xlsx	Metadata for strains used in this study
Tables_S2-S7_statistical_analyses.xlsx	Descriptive and inferential statistics for ARA experiments
Table S9	Primers used to construct tagged strains
Table S10	Diazotroph growth data +/- helper strain

SUPPLEMENTARY FILES DEPOSITED TO FIGSHARE

Figshare: https://doi.org/10.6084/m9.figshare.c.7002321

Zip Files	File Count	Description
ARAdata.zip	11	ARA experiment raw data
R_scripts.zip	9	R scripts and RDS files
TYGS.zip	12	Type (Strain) Genome Server reports
**Individual Files**		**Description**
TOC_figshare.docx	1	Table of Contents, Figshare
~~File_S1_Supplementary_Genomic_Methods.docx~~	1	~~WGS assembly, annotation and taxonomic classification~~
01ARA_cleanDATA.html	1	Notebook of data cleaning scripts
02ARA_getSTATS.html	1	Notebook of statistical analysis scripts
03ARA_reportSTATS.html	1	Notebook of statistical results
04ARA_makeFIGS.html	1	Notebook of figures produced

05ARA_makeTABLES.html	1	Notebook of tables produced
File_S2a_HT1-6_16SrRNA_blastResults.csv	1	HT1-6 16S rRNA blast results
File_S2b_HT1-6_16SrRNA_geneSeq.fasta	1	16S rRNA gene sequence

Total File Count: 43

Supplementary Figure Legends

Figure S1. Screening of extracts of Hawaiian plants for nitrogenase activity using the acetylene reduction assay. Ferns, succulents, and other plants were tested. Of all of the Hawaiian plant sample extracts, HT1 had the highest nitrogenase activity, n=1

Figure S2. Diversity of strains on MGL agar that were in the apparent colony from HT1 on NF-CCM. From left to right: HT1-1, HT1-2, HT1-3, HT1-4, HT1-5; second row: HT1-6, HT1-7, HT1-8, HT1-9, and HT1-10

Figure S3. Representative figure of Comparison of activity between isolated diazotrophs (HT1-6, HT1-9) found within the HT1 community and the mixture of all strains found within the HT1 community, n=3

Figure S4. Comparison of effects on nitrogenase activity of a mix of diazotrophs WP5 and WPB. Compared to synergy strain, WW5, the other yellow pigmented strains had little or even negative impacts on nitrogenase activity, n=1

Figure S5. Set up for physical separation of the diazotrophs and synergists
